# Tunable icephobicity of surface-grown metal–organic frameworks with nanohierarchical texture

**DOI:** 10.1039/d5nr04825g

**Published:** 2026-02-23

**Authors:** Simrandeep Bahal, Jianhui Zhang, Vikramjeet Singh, Prasenjit Kabi, Abbas Heydari, Manish K. Tiwari

**Affiliations:** a Nanoengineered Systems Laboratory, UCL Mechanical Engineering, University College London London WC1E 7JE UK m.tiwari@ucl.ac.uk; b UCL Hawkes Institute, University College London London W1 W 7TS UK; c Manufacturing Futures Lab, UCL Mechanical Engineering, University College London London E20 2AE UK

## Abstract

Metal–organic frameworks (MOFs) have emerged as promising candidates for advanced surface treatments due to their inherent porosity, structural tunability, and functional versatility. Herein, we utilize these unique attributes of MOFs to systematically investigate their potential as passive icephobic surfaces, which is a critical requirement to mitigate ice accretion, which significantly impacts safety and performance across numerous technologies. Specifically, we elucidate how MOFs’ pore size, surface morphology, and chemical functionality synergistically influence ice nucleation temperature and ice adhesion strength. Employing surface-grown MOFs (UiO-66, UiO-67 and MOF-5), we demonstrate that these coatings consistently lowered the median ice nucleation temperature by approximately 4–5 °C and reduced ice adhesion strength by up to two-thirds compared to bare glass. Through a combined approach involving classical heterogeneous nucleation theory and density functional theory simulations, we uncover that the key mechanism driving this icephobic performance is the nanoconfinement effect arising from sub-nanometre pores, which significantly elevates the energy barrier to ice nucleation and minimizes solid-ice contact through the void effect. Furthermore, we identify hydrophobic alkyl silane functionalisation as the most effective chemical strategy to enhance these icephobic properties. These findings provide critical insights into the structure–property relationships that govern icephobic performance, paving the way for the rational design of MOF-based anti-icing coatings for diverse technological applications.

## Abstract 2

We identify the design limits of nanoconfinement in MOF based icephobic coatings. The findings demonstrate that once the pore size is small enough, chemical functionalisation becomes key to further suppressing ice nucleation and ice adhesion.

## Graphical abstract

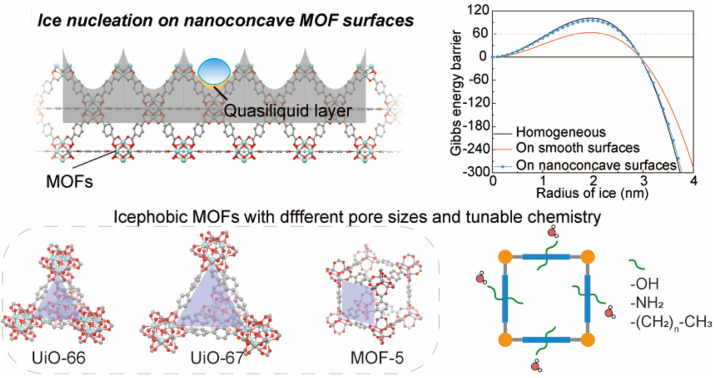

## 1. Introduction

Unwanted ice accretion on surfaces is one of the fundamental problems in nature that poses a serious risk to the safety and operation of a wide range of applications, such as aircrafts,^1,2^ wind turbines,^3–5^ power lines^6,7^ and infrastructure such as buildings and bridges.^8–10^ Considering all these hazardous effects of icing in various applications, it is necessary either to prevent ice formation at the initial stage or to remove ice from various surfaces as soon as it builds up. Over the last few decades, various de-icing strategies have been developed, which include mechanical,^11^ thermal,^12,13^ electrical^14,15^ and chemical methods.^16,17^ However, all these methods are energy intensive, inefficient, expensive and can be corrosive and environmentally unfriendly. In recent years, there has been an endeavour to develop passive icephobic surfaces, which involve surface modifications to achieve one of the following functionalities – to repel incoming water droplets,^18–20^ to delay ice nucleation^21,22^ and to lower the adhesion strength between ice and the underlying substrate.^23–28^ Despite promising icephobic properties, many surfaces suffer from poor durability or environmental resistance, preventing their practical deployment. For example, superhydrophobic surfaces have been shown to repel incoming supercooled water droplets off the surface by virtue of their hierarchical structure and low surface energy.^29–31^ However, these superhydrophobic surfaces tend to fail under high humidity due to frost formation in the textures.^32–34^ Slippery liquid-infused porous surfaces (SLIPS) have shown promise in minimizing the ice adhesion strength.^35–38^ Lubricants, which are usually infused into the micro/nanotextures of the substrate, create a smooth and defect-free interface, which leads to an increased droplet mobility and lowers ice adhesion strength. However, the problem of lubricant depletion by cloaking, evaporation and shear stress limits the practical applications of such surfaces.^39–41^ To overcome this limitation, we designed solid slippery surfaces with nanohierarchical texture using porous materials, including metal–organic frameworks (MOFs) and covalent organic frameworks (COFs). Their intrinsic sub-nanometre porosity not only contributes to mechanical stability but also suppresses ice nucleation by providing nanoscale confinement.^42–45^

Nucleation is the first step in the phase transformation from water to ice according to the classical nucleation theory.^46^ In particular, heterogeneous ice nucleation on solid surfaces typically exhibits a much lower energy barrier than homogeneous ice nucleation, making it more likely to initiate. Hence, inhibiting heterogeneous nucleation is considered the most primitive strategy for the design of icephobic surfaces. The nucleation temperature can be tuned by various strategies such as the use of anti-freeze proteins (AFPs),^47^ peptides^21^ and various ionic salts,^48^ which act by increasing the free energy barrier for ice nucleation at the interface. AFPs can protect organisms living in cold climates from freezing damage by controlling ice formation^49^ and therefore have been introduced to coat substrates for icing control.^50,51^ Within the protein structure, the ice binding face promotes ice nucleation, whereas the non-ice-binding face inhibits it.^51^ However, the extraction of AFPs from natural sources is costly and inefficient, and their inherent thermal instability and susceptibility to denaturation make them unsuitable for long-term or harsh-environment applications. Ionic salts such as NaCl have been employed to eliminate ice accumulation by infusing them into a hydrogel matrix.^52^ However, these salts are easily removed by external factors and require frequent infusion into the matrix.

In addition to the chemical strategies, surface structuring has also emerged as an effective strategy to control ice nucleation.^53–56^ Surface concave nanotextures, which are smaller than the critical nucleus radii (often below 5 nm) can potentially inhibit the nucleation of ice.^57^ It has previously been shown that ice nucleation can be suppressed through the interfacial nano-confinement effect by designing surfaces with precise concave nanotextures.^57^ This confinement has strongly suppressed the stable formation of ice nuclei down to a temperature of −24 °C.^57^ But the fabrication of such surface nanotextures with a few nanometres manufacturing precision is a challenging task. Such nanotextures with confinement effects can be inherently achieved using porous crystalline materials, such as MOFs, which offer sub-nanometre cavities suitable for suppressing heterogeneous ice nucleation. These cavities arise from the highly ordered coordination between metal ions and organic linkers, which form periodic molecular frameworks with tuneable pore sizes.^58–60^ Therefore, the surface-grown MOFs are usually fabricated through bottom-up nanofabrication, such as layer-by-layer (LbL) assembly.^42,61,62^ While previous studies on porous materials have shown nanoconfinement effects,^43,45^ these works typically investigate singular pore geometries, leaving the distinct contributions of geometric confinement, surface structure, and surface chemistry to ice nucleation inhibition unexplored. The inherent tunability of MOFs offers a versatile platform for the rational design of next-generation icephobic surfaces. The modular design of MOFs enables precise control over pore geometry, surface roughness, and interfacial chemistry, making them an ideal platform for systematically studying the factors that govern ice nucleation and ice adhesion.

Hence, in this study, we systematically investigate how crystallographic geometry, surface roughness, and surface functionalisation affect the anti-icing performance of metal–organic frameworks (MOFs). We employed a layer-by-layer (LbL) deposition method to fabricate nanohierarchical films of three distinct MOFs (UiO-66, UiO-67, and MOF-5), enabling independent control over pore size (∼0.6–1.2 nm) and roughness (*via* growth layers). The nanohierarchical structure originates from the intrinsic sub-nanometre pores of the crystalline framework combined with larger nanoscale and mesoscale surface features formed during MOF growth. After systematically varying MOF types and the number of MOF growth layers, we focused on UiO-66 to study the effects of surface functionalisation, including two hydrophilic groups (hydroxyl and amine) and a hydrophobic silane group. By systematically varying pore geometry, surface roughness, and chemical functionality, this study demonstrates a geometric saturation threshold: once the pore size falls below the critical ice nucleus radius, further reduction yields no additional nucleation suppression. Instead, surface chemistry has emerged as the primary determinant in this sub-nanometre regime. The findings provide fundamental insights with practical surface engineering strategies and, to the best of our knowledge, represent the first integrated investigation of these factors in MOF-based icephobic coatings.

## 2. Experimental section

### 2.1. Materials

Microscopic glass slides (75 mm × 25 mm) were purchased from Thorlabs. All the chemicals including zirconyl chloride octahydrate (ZrCl_2_·8H_2_O), zinc nitrate hexahydrate (Zn(NO_3_)_2_·6H_2_O), terephthalic acid, biphenyl-4,4′-dicarboxylic acid, dimethylformamide (DMF), formic acid, acetone, ethanol, isopropanol, *n*-hexane, 3-aminopropyl triethoxysilane (APTES), 2-aminoterephthalic acid, 2,5-dihydroxyterephthalic acid and trichloro-octadecyl silane (OTS) were purchased from Sigma Aldrich. All the chemicals were used as received without further purification. Bottled HPLC-grade water from Sigma Aldrich (HPLC plus 34877) was used for all the icing experiments.

### 2.2. Instrumentation and characterisation

The surface morphologies were imaged using scanning electron microscopy (SEM) and atomic force microscopy (AFM). To do a SEM scan, the specimens were immobilised on a metal stub with a double-sided adhesive carbon tape, followed by a gold film coating. The gold coating was performed using a Leica sputter coater and the thickness of the gold coating was maintained at 5 nm. After gold coating, the samples were observed in the SEM (Carl Zeiss Gemini 360, Germany) at 5 kV voltage. Energy dispersive X-ray spectroscopy (EDS) spectra were obtained for the substrates *via* the attached Oxford EDX system. AFM imaging was performed on the glass substrates using a Bruker Multimode 8-HR AFM with a scanning probe (ScanAsyst-Air) with an Al reflex coated cantilever with a spring constant of 0.4 N m^−1^. All the AFM measurements were performed in non-contact mode. The AFM images were analyzed using the NanoScope Analysis software to evaluate the root mean square roughness (*R*_q_) values. Fourier-transform infrared spectroscopy (FTIR) was performed in the transmission mode with a spectrophotometer (Spectrum Two™, PerkinElmer) in the range of 400–4000 cm^−1^ with a resolution of 2 cm^−1^, accumulating 32 scans. The transparency was assessed using an Orion AquaMate UV-Vis spectrophotometer. The powder X-ray diffraction (XRD) of the MOF film was recorded on a STOE STADI-P spectrometer at ambient temperature, with a tube voltage of 40 kV and a tube current of 40 mA in a stepwise scan mode (5° min^−1^). The advancing water contact angles and contact angle hysteresis were measured using a custom designed goniometer setup.^42^ The setup consisted of an adjustable stage, a retort stand, a syringe pump (World Precision Instruments, Aladdin single-syringe infusion pump), a light source (Thorlabs, OSL2) and a zoom lens (Thorlabs, MVL 7000) fitted to a CMOS camera.^42^ The videos of the droplets were analyzed using ImageJ software to calculate advancing contact angles and contact angle hysteresis.

### 2.3. MOF-film fabrication on glass

The MOF films were synthesised on as-received glass slides using the LbL procedure as reported by Singh *et al.*^42^ Three different types of MOFs, namely UiO-66, UiO-67 and MOF-5 were grown on glass slides. In addition to these three pristine (unfunctionalised) MOFs, UiO-66 was also prepared in hydroxyl-functionalised (UiO-66–OH), amine-functionalised (UiO-66–NH_2_), and alkyl silane-modified (UiO-66–OTS) forms on glass slides. The procedure is mentioned in detail in the following.

#### 2.3.1. APTES functionalisation

Surface functionalisation of as-received glass slides with APTES yielded an amine-terminated self-assembled monolayer, serving as a linker layer for subsequent MOF growth. For this, the glass slide was washed with acetone, followed by isopropanol and DI water and then dried with a stream of dry nitrogen gas. Then, this slide was immersed in 1% *n*-hexane solution of APTES for an hour at room temperature and then dried in an oven for another hour at 120 °C. After drying, the glass slide was rinsed with *n*-hexane to remove the physisorbed molecules and then dried with a stream of nitrogen gas.

#### 2.3.2. Layer by layer growth of MOFs

**Fig. 1:**
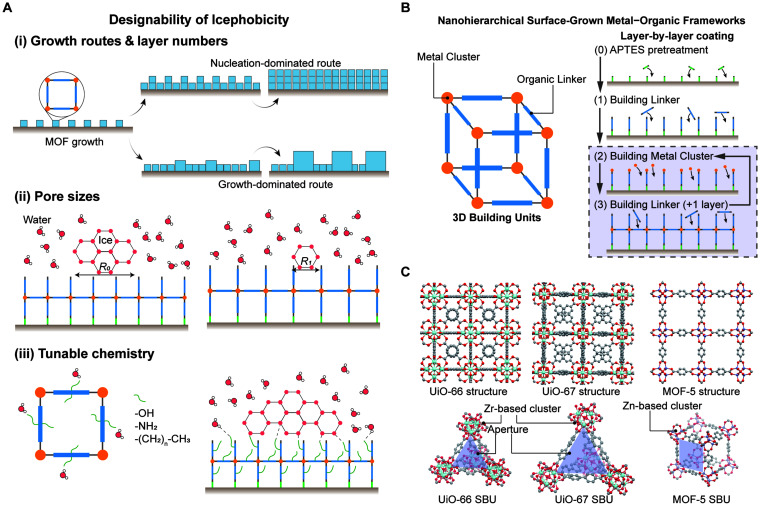
(A) Schematics showing the designing of icephobic MOFs from different aspects, including increasing layer numbers with different growth routes, changing pore sizes, and tuning chemistry with different functional groups. (B) Schematic of building unit structures of MOFs and the LbL technique used to grow MOFs on an APTES functionalised substrate. (C) Schematic representations of the structures of the three different MOFs: UiO-66, UiO-67 and MOF-5. The illustration highlights the differences in their secondary building units (SBUs) with different geometries and pore sizes. All MOF crystal structures were downloaded from the Cambridge Crystallographic Data Centre (CCDC) and visualised using Mercury 2022.2.0.

A 25 mM solution of the respective linker and metal salt was prepared in DMF. The APTES-functionalised glass slides were immersed in the linker solution (25 mM) for 4 hours at 120 °C to get a uniform self-assembly of the linker at the substrate. After 4 hours, the substrate was removed from the linker solution and subjected to bath sonication in DMF for one minute to ensure thorough rinsing. Then, the substrate was immersed in the metal solution (25 mM) for 20 minutes at 120 °C followed by bath sonication in DMF for another one minute. Subsequently, the substrate was immersed in the linker solution (25 mM) for another 20 minutes at 120 °C. This completed one cycle of MOF growth (see Fig. 1A). A suitable number of cycles were repeated to obtain the required growth of the MOF film on the substrate. Finally, once the desired number of layers was grown, the substrate was thoroughly washed with DMF to remove traces of any metal or linker aggregates. The substrate was then dipped in chloroform solution for 48 hours and was then vacuum dried overnight at 100 °C to activate the pores. ZrCl_2_·8H_2_O was used as a metal salt for UiO-66 and UiO-67, whereas Zn(NO_3_)_2_·6H_2_O was taken as a metal salt for MOF-5. Terephthalic acid was used as a linker for pristine UiO-66 and MOF-5, whereas biphenyl-4,4′-dicarboxylic acid was used as a linker for pristine UiO-67.

### 2.4. Synthesis of functionalised MOF surfaces

#### 2.4.1. MOFs with different functional groups

UiO-66 substrates functionalised with two hydrophilic groups (–NH_2_ and –OH) were prepared by the same procedure as mentioned in section 2.3, but with the use of a different linker for each functional group. 2-Aminoterephthalic acid and 2,5-dihydroxyterephthalic acid were used as the linkers for preparing the amine (–NH_2_) and hydroxyl (–OH) functionalised UiO-66 substrate, respectively. UiO-66 was selected as the model MOF platform for systematic surface chemistry modification (–OH, –NH_2_, and –OTS) in order to evaluate the influence of linker functionality on wettability and icephobic behaviour. In addition, a hydroxyl-functionalised MOF-5 substrate was also prepared using 2,5-dihydroxyterephthalic acid as a linker and was subsequently modified with OTS to obtain MOF-5-OTS for durability testing (section 3.6).

#### 2.4.2. Functionalisation of MOFs with silane

Trichloro-octadecyl silane (OTS) was used to alter the wetting properties of the hydroxyl functionalised UiO-66 and MOF-5 substrates. The liquid immersion method was adopted. The hydroxyl terminated MOF substrates were immersed in 1% solution of the OTS in *n*-hexane for an hour and were then placed at 120 °C for another one hour for curing. After that, the substrates were rinsed with *n*-hexane to remove the physisorbed silane molecules and then dried under N_2_ gas and stored for further characterisation studies and tests.

#### 2.4.3. Fabrication of control groups

A glass slide silanised with OTS was prepared to be used as a control for the UiO-66 substrate functionalised with OTS. For that, a liquid immersion method was adopted as explained above. In brief, the glass slide was washed with acetone, isopropanol and DI water and then dried with nitrogen. After that, it was immersed in 1% solution of OTS in *n*-hexane for an hour and was then placed at 120 °C for another hour for curing. The slide was then thoroughly rinsed with *n*-hexane to remove the physisorbed silane molecules and then dried under nitrogen.

#### 2.4.4. Fabrication of linker functionalised glass controls

Glass slides functionalised solely with the linkers – dihydroxyterephthalic acid and 2-aminoterephthalic acid – were also prepared as control substrates to compare with UiO-66 substrates functionalised with hydrophilic groups (–NH_2_ and –OH). For that, the cleaned bare glass substrates were treated with APTES to introduce reactive amine groups. The APTES-functionalised glass substrates were subsequently immersed in a 25 mM solution of the respective organic linker in DMF solution at 120 °C for 4 hours. This procedure facilitated uniform self-assembly and covalent attachment of the linkers onto the amine-modified glass substrate. The resulting surfaces (glass–OH and glass–NH_2_) served as a non-porous chemical analogue for comparison with their corresponding MOF-coated counterparts (UiO-66–OH and UiO-66–NH_2_). Table 1 summarises the MOF coatings and their corresponding control substrates tested and characterised in this work.

**Table 1:** Summary of MOF coatings tested and characterised in this work

MOF type	Functionalisation	Control substrate	Characterisation/tests performed
UiO-66	None (pristine)	Bare glass	FTIR, PXRD, SEM-EDS, AFM, UV-Vis, CA, ice nucleation, ice adhesion
UiO-67	None (pristine)	Bare glass	FTIR, PXRD, SEM-EDS, AFM, UV-Vis, CA, ice nucleation, ice adhesion
MOF-5	None (pristine)	Bare glass	FTIR, PXRD, SEM-EDS, AFM, UV-Vis, CA, ice nucleation, ice adhesion
UiO-66–OH	Hydroxyl linker	Glass–OH	FTIR, CA, ice nucleation, ice adhesion
UiO-66–NH_2_	Amine linker	Glass–NH_2_	FTIR, CA, ice nucleation, ice adhesion
UiO-66–OTS	OTS on UiO-66–OH	Glass–OTS	CA, ice nucleation, ice adhesion, tape peel test
MOF-5–OTS	OTS on MOF-5-OH	Glass–OTS	Ice adhesion, tape peel test

### 2.5. Ice nucleation temperature and ice adhesion measurement

Ice nucleation temperature and ice adhesion strength were measured using a custom benchtop setup based on a Peltier cooling element coupled with a temperature control system (see details in the SI). The substrate was placed in thermal contact with the Peltier element, and its temperature was reduced at a controlled cooling rate (1 °C min^−1^). The temperature was monitored using a type K thermocouple and recorded with a data acquisition system.

For ice nucleation experiments, around 30 water droplets (2 μL each) were deposited on the substrate kept at room temperature. A gentle nitrogen flow was maintained to prevent frost formation during the experiments. The temperature of the substrate was then reduced from room temperature at a cooling rate of 1 °C min^−1^ until all the droplets were frozen. Droplet freezing was recorded using a webcam to detect the freezing of individual droplets. Frozen droplets were identified based on their characteristic white appearance due to light scattering. The ice nucleation temperature of each droplet was extracted from the video, and the fraction of frozen droplets, *f*_ice_, was calculated as:1
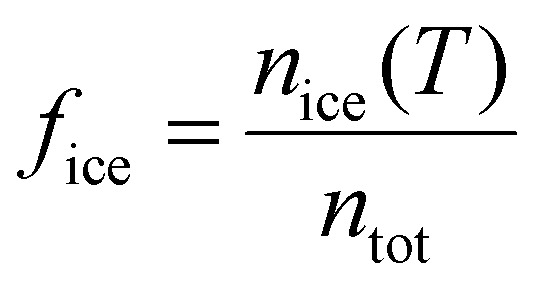
where *n*_ice_(*T*) is the number of frozen droplets at temperature *T* and *n*_tot_ is the total number of droplets. Each measurement was repeated in triplicate. Since both the samples and droplets were slowly cooled from room temperature, and the MOF coatings were extremely thin, the effect of heat transfer through the coating on the observed ice nucleation temperature was considered negligible. This assumption is supported by a small Biot number (<0.01) and a thermal diffusion time (10^−8^–10^−6^ s) several orders of magnitude shorter than the experimental cooling time (>10^3^ s), indicating negligible temperature gradients across the coating.

For ice adhesion measurements, a cylindrical cuvette (8 mm inner diameter and 11 mm outer diameter) was placed on the substrate and filled with water. The system was cooled to −20 °C and held for 1 hour to ensure complete freezing. A horizontal shear force was applied to the cuvette using a motorised stage, and the force gauge was moved at 0.02 mm s^−1^. The peak force *F* required to detach ice was recorded, and the ice adhesion strength *τ* was calculated as:2
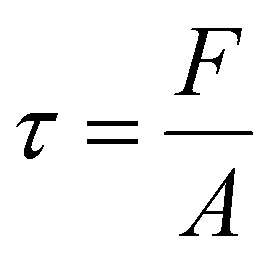
where *A* is the contact area of the cuvette. Each condition was tested on at least three samples to ensure reproducibility. A representative force–time curve for the ice adhesion measurement is shown in Fig. S1 (SI). The maximum force recorded before ice detachment was used to calculate the ice adhesion strength, normalised by the ice-substrate contact area.

### 2.6. Tape peel test

A tape peel test was applied to evaluate the mechanical durability of the coatings, using pressure-sensitive and strong adhesive tape (3 M VHB™ tape 5952 with an adhesive peel strength of 3900 N m^−1^). The tape was applied on the horizontally placed MOF coated substrates. To ensure intimate contact and eliminate trapped air, a 2 kg stainless steel roller was passed once over the tape. After a dwell time of 60 seconds, the tape was peeled back on itself in a single, steady motion, constituting one test cycle. The contact angle measurements were performed after each cycle. The process was repeated, and a fresh piece of tape was used in each cycle. This tape-peel procedure is a common durability assessment that has been extensively used in prior works to evaluate the adhesion and stability of icephobic coatings.^42–44^

### 2.7. Statistical analysis

All data are expressed as the mean ± standard deviation from more than three independent experiments or samples. Statistical analysis was performed using one-way ANOVA followed by Tukey's multiple comparison test. Differences were considered statistically significant at *p* < 0.05 (*p < 0.05, **p < 0.01, ***p < 0.001, ****p < 0.0001), while NS indicates no significant difference between the compared groups.

## 3. Results and discussion

### 3.1. Design of icephobic surface-grown MOFs

The aim of this study is to tune the anti-icing behaviour of MOF-coated substrates by leveraging the designability of MOFs through controlled variations in the changes of pore sizes, roughness and chemical functionalisation. As illustrated in Fig. 1A, we designed a modular strategy to independently control these three parameters. Surface roughness was tuned by varying the number of growth cycles in an LbL assembly process. Additionally, differences in crystal growth mechanisms across various MOFs *viz*. nucleation-dominated *vs*. growth-dominated further contributed to variations in surface texture. Pore size was adjusted by selecting MOFs with different metal nodes and organic linkers, while surface chemistry was tailored by introducing various functional groups on the organic ligands, thereby altering wettability and interfacial interactions with ice.

To implement this strategy, nanostructured MOF films were grown on amine-functionalised glass substrates using a LbL approach (Fig. 1B). The LbL method was chosen to precisely control film thickness and surface morphology and to minimize aggregation-induced roughness commonly observed in spray-based deposition methods. This approach also enabled the formation of nanostructured MOF films covalently bonded on substrates while preserving the intrinsic sub-nanometre pore structure of individual MOF crystals. Three MOFs – UiO-66 (pore size ∼0.6 nm), MOF-5 (pore size ∼0.8 nm), and UiO-67 (pore size ∼1.2 nm) – were chosen for their distinct pore sizes and framework topologies (Fig. 1C). UiO-66 and UiO-67 are zirconium-based frameworks, while MOF-5 is zinc-based.^63,64^ These MOFs represent structurally diverse frameworks for systematically exploring structure–function relationships in ice nucleation and ice adhesion measurements.

### 3.2. Chemical and surface characterisation

**Fig. 2:**
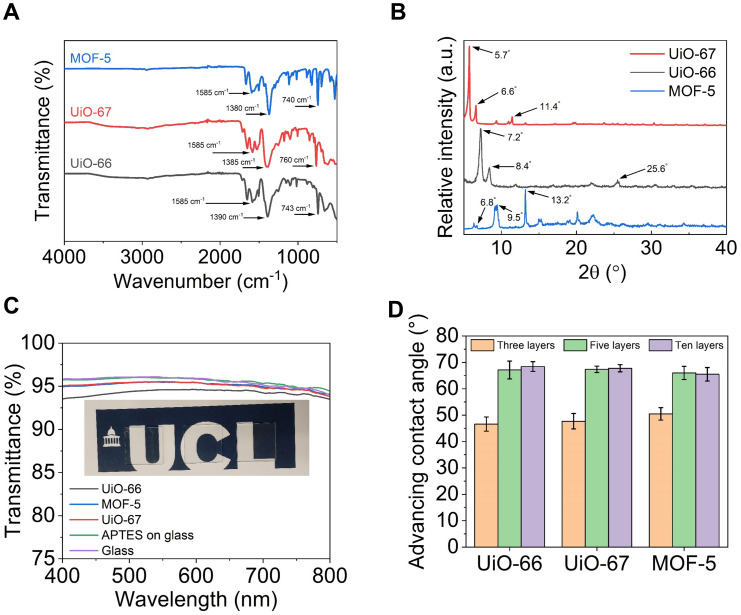
Characterisation of the nanohierarchical MOFs grown on glass through the LbL process. (A) FTIR spectra of the three different surface grown MOFs. (B) PXRD graph of the three different MOF films. (C) Transmittance of the MOF-coated glass substrates measured using UV-Vis spectroscopy in a five-layer configuration. Inset: Photographs of the three MOF-coated glass slides (left to right: UiO-66, UiO-67, and MOF-5) in the five-layer configuration, highlighting their optical transparency. (D) Advancing water contact angles when the number of MOF layers was three, five and ten.

The chemical structure of the three pristine (without any functionalisation) MOFs grown on surfaces was confirmed by FTIR with characteristic peaks which confirmed the successful formation of the MOFs as shown in Fig. 2A. The FTIR spectrum of MOF-5 displayed two peaks at 1380 cm^−1^ and 1585 cm^−1^ which were ascribed to the symmetric and asymmetric stretching vibration of the carboxylate (COO^−^) groups in the terephthalic acid linker. This confirmed the successful coordination of the carboxylate groups of the terephthalic acid linker to the zinc oxide (Zn_4_O) clusters. The peak at 740 cm^−1^ was attributed to the C–H out-of-plane bending vibrations of the aromatic ring in the linker, indicating that the aromaticity of the terephthalic acid was preserved during the synthesis. Additionally, the vibration bands between 500 and 700 cm^−1^ were associated with the Zn–O stretching vibrations within the Zn_4_O clusters, which further validated the formation of the tetrahedral zinc oxide secondary building units. These results were consistent with the FTIR spectra of MOF-5 reported in the literature.^65,66^ For UiO-66, the observed peaks at 1390 cm^−1^ and 1585 cm^−1^ represented the symmetric and asymmetric stretching vibrations of the carboxylate groups, respectively, thereby confirming the successful coordination of the carboxylate groups of terephthalic acid with the zirconium Zr_6_O_4_(OH)_4_ clusters. The peak at 743 cm^−1^ was associated with the C–H out-of-plane bending vibrations of the aromatic ring, while the vibration bands observed between 500 and 700 cm^−1^ were characteristic of the Zr–O stretching, which verified the formation of the zirconium oxide clusters. These observations aligned with the reported FTIR spectra of UiO-66 in the literature.^67–69^ For UiO-67, the symmetric and asymmetric stretching vibrations of the carboxylate groups were observed at 1385 cm^−1^ and 1585 cm^−1^, respectively, similar to that of UiO-66. Additionally, the peak at 760 cm^−1^ was attributed to the C–H out-of-plane bending vibrations of the biphenyl linker, and vibration bands between 500 and 700 cm^−1^ corresponded to Zr–O stretching, validating the formation of the zirconium oxide clusters. These results were in agreement with the reported FTIR spectra of UiO-67 in the literature.^70^ The crystalline structures of the MOF films grown on glass substrates were confirmed using powder XRD as shown in Fig. 2B. For UiO-66, characteristic diffraction peaks were observed at 2*θ* ≈ 7.2°, 8.4° and 25.6°.^69^ Similarly, for UiO-67, peaks at 2*θ* ≈ 5.7°, 6.6° and 11.4° were detected, which confirmed its larger unit cell due to the longer biphenyl linker.^70^ For MOF-5, the diffraction pattern exhibited characteristic peaks at 2*θ* ≈ 6.8°, 9.5° and 13.2°.^65^ All XRD patterns were consistent with those reported in the literature.

The optical properties of the three surface grown MOFs on glass were evaluated using UV-Vis spectroscopy, as shown in Fig. 2C. The results demonstrated that all three surface grown MOFs exhibited high optical transparency with transmittance values ≥93% across the visible spectrum, 400–800 nm. This indicated that the MOF films possessed smooth, nanohierarchical textures with minimal surface scattering, suggesting uniform film formation across the substrate. The wettability of the MOF-coated glass substrates was evaluated by measuring the advancing water contact angle (*θ*_A_) for samples with three, five, and ten layers of UiO-66, UiO-67 and MOF-5 (Fig. 2D). All surfaces exhibited hydrophilic behaviour, with contact angles consistently below 90°. Due to strong contact line pinning during droplet withdrawal, receding contact angles and contact angle hysteresis could not be reliably quantified. At three layers, contact angles were low and comparable (46–51°) across all MOFs, indicating similar surface wettability regardless of the pore structure or chemistry. As the number of layers increased to five, *θ*_A_ increased to ∼65–67°, which may be attributed to the enhanced surface roughness and more complete coverage of MOFs (SI Fig. S7). The *θ*_A_ remained nearly constant (65–68°) beyond ten layers, suggesting that surface wettability had saturated due to full MOF coverage.

### 3.3. Surface morphology with different layers

**Fig. 3:**
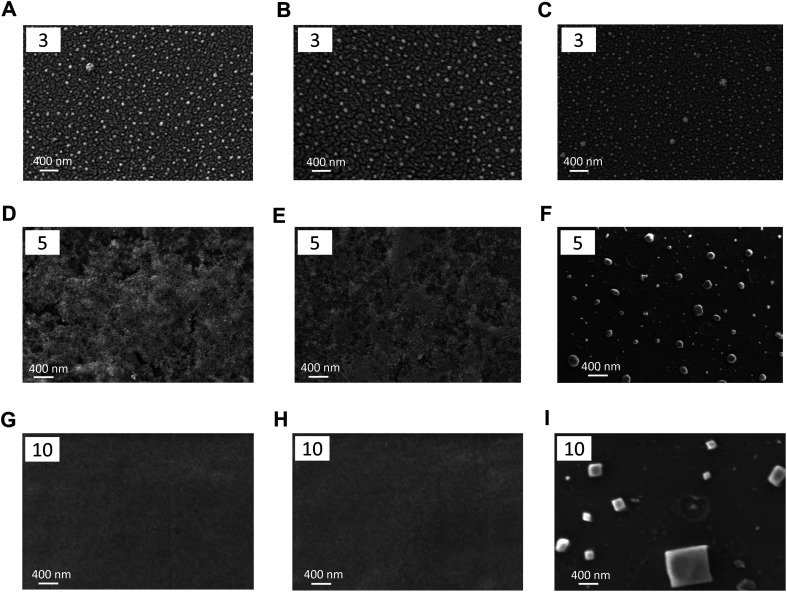
SEM images showing the surface morphology of UiO-66, UiO-67 and MOF-5 films grown on glass substrates *via* the LbL technique. Rows correspond to increasing numbers of layers – three layers (A–C), five layers (D–F) and ten layers (G–I). Columns represent different MOFs: UiO-66 (left panel), UiO-67 (middle panel), and MOF-5 (right panel). The number of layers used for each configuration is indicated at the top-left corner of each SEM image for clarity. The images illustrate the morphological evolution from discrete particles at lower layers to increasingly interconnected structures at a higher number of layers.

**Fig. 4:**
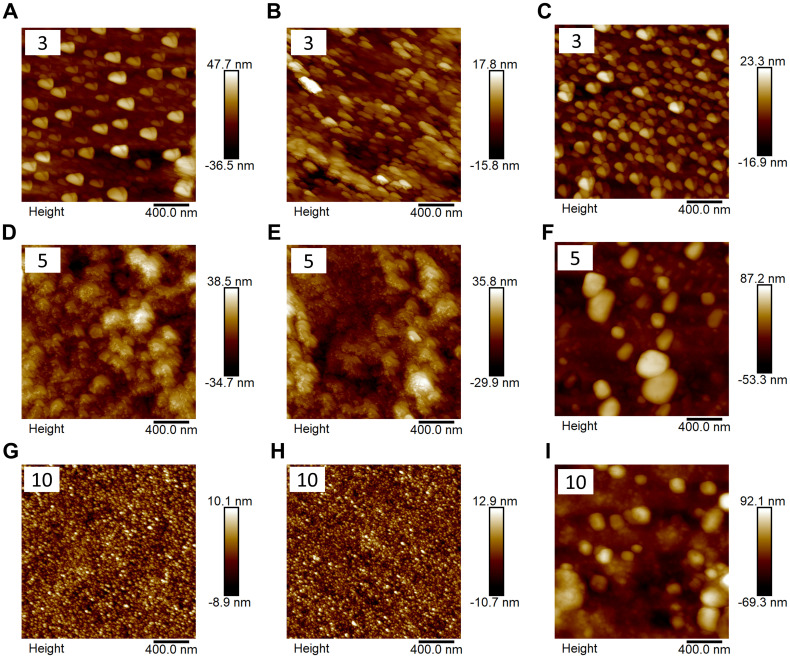
AFM topography images corresponding to UiO-66, UiO-67 and MOF-5 films grown on glass substrates *via* the LbL technique. Rows indicate increasing numbers of layers – three layers (A–C), five layers (D–F) and ten layers (G–I). Columns correspond to UiO-66 (left panel), UiO-67 (middle panel), and MOF-5 (right panel). The number of layers used for each configuration is indicated at the top-left corner of each AFM image for clarity. The AFM images quantitatively capture changes in surface roughness and morphology as the number of layers increases, complementing the SEM observations.

The surface morphology of MOF-coated glass substrates with varying layer numbers was characterised using SEM and AFM, as shown in Fig. 3 and 4, respectively. When the number of layers was three, all three MOFs exhibited a discontinuous “island-like” growth pattern consisting of dispersed nanoparticle clusters rather than a continuous film (Fig. 3A–C and 4A–C). Despite incomplete surface coverage, the particles appeared uniformly distributed across the substrate. This early-stage island-type morphology is a characteristic of the LbL assembly method.^71^ The cross-sectional height profiles were recorded to compare the surface roughness of bare glass and MOF-coated substrates. At three layers, all MOF coatings exhibited noticeably rougher surfaces than bare glass (Fig. S3 and S4, SI). The root mean square roughness (*R*_q_) values for UiO-66, UiO-67 and MOF-5 were found to be 12.6 nm, 11.8 nm, and 6.6 nm, respectively (Fig. S2), all significantly higher than that of the bare glass substrate (0.54 nm). Under the partial coverage regime, the exposed glass areas could negatively impact the overall surface hydrophilicity (Fig. 2D) and anti-icing performance of the MOF coatings.

Upon increasing the layer number to five, the existing MOF particles continued to grow and merged with adjacent particles, gradually forming a more continuous film and resulting in significantly improved surface coverage (Fig. 3D–F and 4D–F). With further MOF deposition, interparticle gaps decreased, and the coating became more uniform. For UiO-66 and UiO-67, the surface exhibited denser and more interconnected structures (Fig. 3D and E, 4D and E), although cracks and voids were still occasionally observed between particles. This morphology suggests that the growth of UiO type MOFs follows a nucleation-dominated Frank–van der Merwe mode, wherein a low nucleation energy barrier facilitates the frequent formation of new nuclei.^71,72^ In contrast, MOF-5 displayed larger, well-defined particles with distinct boundaries (Fig. 3F and 4F), indicating a likely “layer plus island” Stranski–Krastanov growth mode, in which existing nuclei grow rapidly while new nucleation events become less frequent.^73^ Although the SEM images for the five-layer configuration of MOF-5 (Fig. 3F) show the presence of large, well-defined particles, these features did not form isolated particles on bare glass. A continuous underlying MOF-5 layer had already developed during the initial LbL deposition cycles, upon which these larger crystallites nucleated and grew vertically. This dual morphology, consisting of a uniform base film with superimposed faceted domains, is a characteristic of the layer-plus-island (Stranski–Krastanov) growth mode.^73^ Compared to the three-layer coatings, the root mean square roughness (*R*_q_) values of all three MOFs increased to 17.6 nm for UiO-66, 15.7 nm for UiO-67 and 22.5 nm for MOF-5 (Fig. S2), consistent with the denser and more interconnected surface morphology observed in SEM. Among these, MOF-5 exhibited the highest surface roughness, reflecting its more prominent particle growth at this stage. Distinct morphological features between UiO-type MOFs and MOF-5 were reflected in the difference in the distribution of roughness radius of curvature, as shown in Fig. S5. Continuous LbL growth cycles facilitated improved surface uniformity and integrity.^71^ Correspondingly, the enhanced surface coverage and roughness led to increased water contact angles (see Fig. 2D).

When the number of layers was increased to ten, the differences between the two growth modes became more pronounced. SEM images revealed that UiO-66 and UiO-67 coatings evolved into more continuous and smoother films, indicating extensive particle coalescence and near-complete surface coverage (Fig. 3G and H). In contrast, MOF-5 showed well-faceted, large cubic crystals with clearly defined crystal planes (Fig. 3I), indicative of substantial vertical overgrowth and a distinct morphological evolution. This morphology further confirmed that the MOF-5 film was composed of a continuous underlying layer supporting vertically overgrown cubic crystallites, consistent with the progression of the layer-plus-island growth mechanism observed at earlier deposition stages. The cross-sectional height profiles at five and ten layers (Fig. S6 and S7) quantitatively reflect the morphological evolution associated with the two distinct film formation mechanisms. The root mean square roughness (*R*_q_) values for UiO-66 and UiO-67 decreased significantly to ∼2.7 nm and 3.3 nm, respectively, suggesting a transition from island-type to continuous and smooth films. In contrast, MOF-5 retained a high roughness (∼23 nm), consistent with the large crystal features observed in SEM. Despite the notable morphological changes from five to ten layers, the advancing water contact angles of all three MOFs remained nearly unchanged compared to the five-layer samples (Fig. 2D), indicating that the surface was already sufficiently covered by MOFs at five layers and that further roughness evolution had little additional influence on surface wettability.

Elemental composition and distribution were examined using energy-dispersive X-ray spectroscopy (EDS), as shown in Fig. S8 and S9. The uniform detection of Zr and Zn confirms the successful deposition of UiO-type MOFs and MOF-5, respectively. The Si signal observed in these spectra originates entirely from the underlying glass substrate. This is further evidenced by the complementary distribution of the elements. The Si signal is notably attenuated in regions densely covered by MOF crystals, confirming that it arises from the support beneath.

### 3.4. Ice nucleation temperature and adhesion measurements

#### 3.4.1. Influence of the assembly layer number

**Fig. 5:**
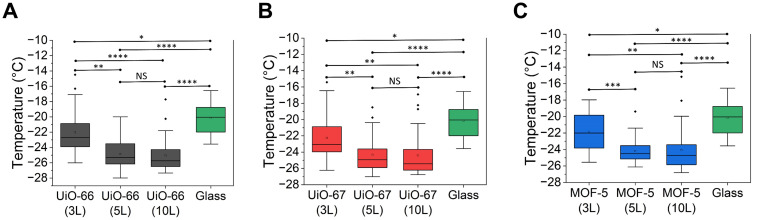
Ice nucleation temperature of the MOF-coated substrates with different layers. (A) UiO-66, (B) UiO-67 and (C) MOF-5. For each material, box-and-whisker plots compare the ice nucleation temperatures obtained on coatings with three (3L), five (5L) and ten layers (10L) of MOF growth and compare them with those measured on bare glass (boxes = 25^th^–75^th^ percentiles; centre lines = medians; whiskers = 5^th^–95^th^ percentiles). The centreline of each boxplot indicates the median ice nucleation temperature, equal to T_50_, which is the temperature at which 50% of the droplets were frozen. *p < 0.05, **p < 0.01, ***p < 0.001 and ****p < 0.0001. NS denotes that the difference between the indicated groups is not statistically significant.

The icephobic performance of these MOF-based coatings consisting of UiO-66, 67 and MOF-5, was evaluated by measuring the ice nucleation temperatures of water droplets on substrates with different growth layers. Bare glass was included as a control. As shown in Fig. 5A–C, boxplots were used to visualize the distribution of ice nucleation temperatures for each substrate. Across all three MOF-coated substrates, the median ice nucleation temperature decreased as the number of layers increased from three to five but remained nearly unchanged when the number of layers was further increased to ten layers. This trend indicated that the reduction in ice nucleation temperature reached a saturation point beyond five layers. Notably, the median ice nucleation temperature on all three MOF-coated substrates, regardless of the layer number, was consistently lower than that on bare glass, which showed the intrinsic icephobicity of these MOFs, attributable to their nanohierarchical surface texture even without post-functionalisation.

This observed reduction in ice nucleation temperature from three to five layers was primarily due to the improved surface coverage of MOF coatings. SEM images (Fig. 3A–C) showed that at three layers, all MOF films exhibited incomplete coverage with visible gaps between particles, allowing direct contact between water droplets and the underlying hydrophilic glass, thereby reducing the effectiveness of the coating. Upon increasing to five layers, SEM imaging (Fig. 3D–F) revealed more densely packed and interconnected particle networks, significantly reducing substrate exposure and enhancing ice nucleation inhibition. Further increasing the number of layers to ten (Fig. 3G–I) did not lead to a noticeable improvement in surface coverage, indicating a saturation point beyond which additional layers no longer enhanced performance, correlating with the unchanged ice nucleation temperatures.

Although the morphological differences between five-layer and ten-layer MOF coatings were reflected in variations in surface texture and roughness, the median ice nucleation temperature of water droplets on these surfaces remained unchanged. This indicates that, once the substrate is sufficiently covered by the MOF coating, further modulation of surface roughness at the nano- or microscale has minimal effect on ice nucleation behaviour.^74,75^ A similar trend was observed when comparing our pristine MOF coatings with nanohierarchical texture to previously reported MOF-based superhydrophobic coatings with micro/nanotextures.^45^ Although those surfaces exhibited a Cassie state while our droplets were in a Wenzel state, the median ice nucleation temperatures are comparable. These results strongly suggest that the anti-icing performance of MOF-coated substrates under conditions of full surface coverage is governed primarily by the intrinsic sub-nanometre pore structures of the MOFs,^43^ rather than by surface topography. The pore sizes of UiO-66 (0.6 nm), MOF-5 (0.8 nm) and UiO-67 (1.2 nm) are significantly smaller than the critical ice embryo radius (2.3–4.4 nm in the temperature range examined, −10 °C to −20 °C, see details in the SI) and thus cannot accommodate a stable ice nucleus. As a result, water molecules that enter these pores remain in a highly confined state, which suppresses ice nucleation. This nanoconfinement effect^57^ provides a robust mechanism for freezing point depression, independent of mesoscale or microscale roughness modulation.

#### 3.4.2. Influence of pore size and geometry

**Fig. 6:**
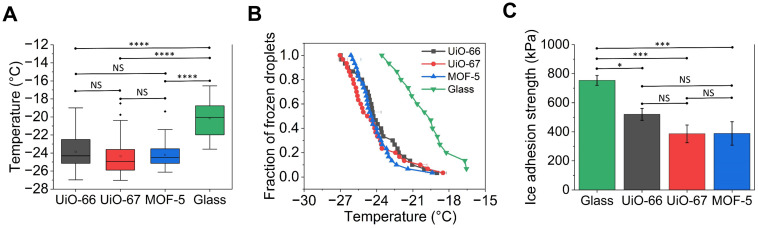
Influence of the pore size and geometry of MOFs on the icephobic performance using MOF-coated substrates with five growth layers. (A) Box-and-whisker plots of ice nucleation temperatures for UiO-66, UiO-67 and MOF-5 compared with bare glass (boxes = 25^th^–75^th^ percentiles; centre lines = medians; whiskers = 5^th^–95^th^ percentiles). (B) Freezing spectra (fraction of frozen droplets *versus* temperature) highlighting the leftward shift for all the MOF-coated substrates relative to bare glass. (C) Ice adhesion strength measured at −20 °C for the three MOF-coated substrates and bare glass. *p < 0.05, **p < 0.01, ***p < 0.001 and ****p < 0.0001. NS denotes that the difference between the indicated groups is not statistically significant.

Next, the influence of the pore size and geometry on ice nucleation temperature and ice adhesion strength was investigated by comparing the icephobic performance of UiO-66, 67 and MOF-5 (Fig. 6). It is evident that all three different MOF-coated substrates had significantly reduced both the median ice nucleation temperature (Fig. 6A) and the onset freezing temperature (Fig. 6B) – defined as the temperature at which the first droplet had frozen compared to that on the bare glass substrate. On bare glass, the median ice nucleation temperature was ∼−20 °C, whereas on UiO-66, 67 and MOF-5, the values were around −24.3 °C, −24.8 °C and −24.4 °C, respectively. The onset freezing temperature on bare glass was around −16.5 °C, whereas on the MOF-coated substrates, it was slightly lower, between −18 °C and −19 °C. Similarly, the last few droplets on MOF-coated substrates froze at a lower temperature (∼−26 °C to −27 °C) compared to −23 °C on bare glass, further highlighting the ice nucleation inhibition effect of these coatings. However, no statistically significant differences were observed in the representative mean ice nucleation temperatures among the three MOF-coated substrates as confirmed by the ANOVA test (Fig. 6A). This suggests that differences in the pore size and geometry among the MOFs did not play a dominant role in governing ice nucleation.

We attribute this phenomenon to the fact that these MOFs possess similar sub-nanometre pore sizes. Since all the pore sizes are considerably smaller than the critical ice-embryo radius *r*_c_ (2.3–4.4 nm in the temperature range examined; details are given in the SI), the resulting nanoconfinement effect increases the free-energy barrier for heterogeneous nucleation to a similar extent on each of the three MOF-coated surfaces, thereby suppressing ice formation compared with glass but yielding comparable ice nucleation temperatures.^43,57^ The interfacial behaviour of water on MOF surfaces may also be influenced by specific interactions between water molecules and the organic linkers or metal clusters of the frameworks. Such interactions – hydrogen bonding, coordination effects, or local ordering – can modulate the orientation and dynamics of water molecules near the surface, potentially stabilizing or destabilizing ice-like clusters.^76,77^ As such, the comparable ice nucleation temperatures observed across chemically similar MOFs may arise not only from similar pore sizes but also from convergent intermolecular environments that affect ice nucleation. Therefore, for deeper insights that underpin the nanoconfinement effect, molecular-level simulations were undertaken (section 3.5).

The ice adhesion strength on these three MOF-coated substrates was further evaluated and compared with bare glass, as shown in Fig. 6C. It can be observed that all three MOF-coated substrates significantly reduced the ice adhesion strength compared to that on bare glass. This reduction was statistically significant and was attributed to the nanohierarchical, highly porous architecture of MOF-coated surfaces which decreased the effective molecular-level contact area between ice and the solid components of the MOFs, namely, the organic linkers and metal clusters.^44,78–80^ It is important to clarify that the wetting state of water on these MOF coatings exists in a hybrid regime across different length scales. At the mesoscale, the droplets likely penetrated the roughness features arising from the packing of MOF particles, resulting in a predominantly Wenzel-type wetting behaviour. This interpretation was supported by the strong contact-line pinning and very low receding angles observed on pristine MOF coatings, which are characteristic of fully wetted hydrophilic textures rather than a Cassie–Baxter state. Importantly, the reduction in the ice adhesion strength was not attributed to macroscopic air entrapment beneath the droplet but instead to a nanoscale void effect associated with the intrinsic sub-nanometre porosity of the MOF frameworks. Water and ice could not conformally penetrate these primary pores due to the large free-energy barrier imposed by nanoconfinement.^43,45,57^ As a result, although the droplet appears macroscopically wetted and pinned, the effective molecular-level ice–solid contact area was substantially reduced because contact occurred only on the solid framework struts (metal clusters and organic linkers), with the internal pores remaining non-contacting voids. This reduction in true interfacial contact area lowered the work of adhesion and facilitated interfacial crack initiation, thereby explaining the decreased ice adhesion strength despite the absence of a Cassie wetting state.

Although UiO-67 and MOF-5 exhibited slightly lower mean adhesion values compared to UiO-66, these differences were not statistically significant. This indicates that varying pore geometries in the sub-nanometre regime have a negligible impact on macroscopic ice adhesion. The persistent adhesion across all three pristine MOFs is likely driven by their inherently high surface energy and similar roughness scales, rather than specific pore dimensions. Consequently, to achieve a substantial reduction in ice adhesion, the strategy must shift from geometric variation to chemical functionalisation. This motivates the next section, where we systematically tune surface chemistry through linker modification.

It should be noted that these sub-nanometre pore sizes of MOFs correspond to the intrinsic pores within the crystalline frameworks of UiO-66, 67 and MOF-5, which arise from the coordination geometry of their metal clusters and organic linkers. These framework pores (0.6–1.2 nm in diameter) are inherent structural features of the MOFs and remain unchanged with increasing numbers of LbL deposition cycles. Increasing the number of layers only improves the film continuity and surface coverage, as explained in section 3.3, but does not alter the internal pore dimensions. As a result, while all three MOFs share similar intrinsic pore sizes, their overall roughness and surface morphology evolve with the number of layers.

#### 3.4.3. Effect of linker chemistry

Next, the influence of surface chemistry on icephobicity was systematically investigated by comparing pristine UiO-66 with chemically functionalised variants, including UiO-66–NH_2_ (amine groups), UiO-66–OH (hydroxyl groups), and UiO-66–OTS (modified with hydrophobic OTS silane groups). The successful synthesis of UiO-66–NH_2_ and UiO-66–OH through LbL growth with five cycles was confirmed by FTIR spectroscopy (Fig. S11, SI). Glass substrates functionalised with an APTES self-assembled monolayer followed by grafting of the MOF linker molecules were used as control samples. These surfaces replicate the chemical functional groups present on the MOF-coated substrates but lack their characteristic nanostructured porosity, providing a smooth, non-porous surface for comparison.

**Fig. 7:**
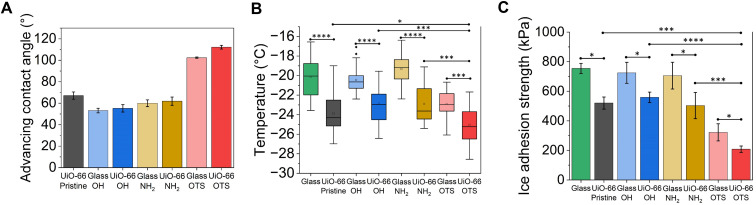
Effect of surface functional groups of UiO-66 on the icephobic performance. (A) Advancing water contact angles. (B) Box-and-whisker plots of ice nucleation temperatures (boxes = 25^th^–75^th^ percentiles; centre lines = medians; whiskers = 5^th^–95^th^ percentiles). (C) Ice adhesion strength measured at −20 °C for the various functionalised substrates, pristine substrate and bare glass. *p < 0.05, **p < 0.01, ***p < 0.001 and ****p < 0.0001.

Fig. 7A presents the advancing contact angle (*θ*_A_) measured on substrates coated with pristine UiO-66, UiO-66–OH, UiO-66–NH_2_ and UiO-66–OTS, as well as on glass substrates functionalised with the corresponding MOF linkers. The pristine UiO-66 exhibited an advancing contact angle of approximately 65°, while the introduction of hydroxyl and amine groups reduced the advancing contact angles to the range of 50–60°, indicating enhanced surface hydrophilicity. The hydrophilic MOF-coated substrate and its corresponding linker functionalised glass counterpart showed nearly identical advancing contact angles. However, the receding contact angles could not be measured due to strong contact-line pinning during droplet withdrawal. Consequently, for these pristine and hydrophilic UiO-66 substrates, only advancing contact angles are reported. In contrast, OTS modification significantly enhanced hydrophobicity. UiO-66–OTS exhibited a higher advancing contact angle (∼112°) and reduced hysteresis (∼11°) compared to OTS-glass (∼102° and ∼20°, respectively; Fig. S10). This enhanced droplet mobility suggests a synergistic effect between the nanohierarchical roughness and low-surface-energy alkyl chains, effectively minimizing the solid–liquid contact area and improving slipperiness.^42^

Fig. 7B compares the ice nucleation temperatures of water droplets on these various functionalised substrates. Substrates coated with UiO-66, including pristine, hydroxyl-functionalised, amine-functionalised, and alkyl silane-modified forms, all showed a consistent shift in the freezing spectrum to lower temperatures. This was in contrast to glass substrates with identical surface chemistries but no MOF coating. The median ice nucleation temperature on bare glass was approximately −20 °C and it decreased to about −24 °C on the substrate coated with pristine UiO-66. A similar ∼2 to 3 °C downward shift was observed with hydroxyl or amine functionalised UiO-66 substrates, even though the corresponding linker-functionalised glass slides displayed identical wettability to the MOF coatings. Such a reduction in ice nucleation temperature is typically associated with orders of magnitude difference in the freezing delay time, as reported in prior studies.^57^ This highlighted the contribution of the MOF's intrinsic sub-nanometre porosity. These experimental results further support the claim that UiO-66 exerts a pronounced nanoconfinement effect. By inhibiting the stabilisation of a critical ice nucleus, the porous MOF coating increases the free-energy barrier for heterogeneous ice nucleation compared to non-porous glass substrates. Among all surface chemistries, UiO-66–OTS exhibited the strongest suppression of ice nucleation, with a median ice nucleation temperature of ∼−25 °C, significantly lower than those of other MOFs and all glass controls. This enhanced performance is attributed to the synergistic effect of the nanoconfinement provided by the MOF framework and the increased surface hydrophobicity imparted by the long-chain alkyl silane, which together raise the free-energy barrier for ice nucleation.

Fig. 7C compares the shear ice adhesion strengths measured at −20 °C. Across all surface chemistries tested, UiO-66-coated substrates markedly reduced ice adhesion. UiO-66 with hydrophilic terminations reduced ice adhesion strength by roughly one-third compared to its glass counterparts, and the pristine UiO-66 substrate also showed a comparable reduction despite exhibiting a higher contact angle. Notably, UiO-66–OTS exhibited the lowest ice adhesion strength, about two-thirds lower than that of bare glass and significantly lower than those of all other MOFs and glass controls. The superior icephobic performance of the UiO-66-coated surfaces, even when their macroscopic wettability was similar to that of the corresponding glass controls, was attributed to their nanohierarchical porosity.^43,57^ The porous framework reduced the actual solid–ice contact area, thereby lowering the shear force required for ice detachment. The added benefit of OTS functionalisation arose from the flexible, low-surface-energy alkyl chains, which further diminished interfacial adhesion and promoted crack propagation. These findings reinforce the conclusions drawn from the ice nucleation experiments: the porous architecture of the MOF coating is the dominant factor governing icephobic behaviour, while hydrophobic surface chemistry provided an additional enhancement, most pronounced for OTS modification.

### 3.5. Insights into nucleation and molecular simulations

**Fig. 8:**
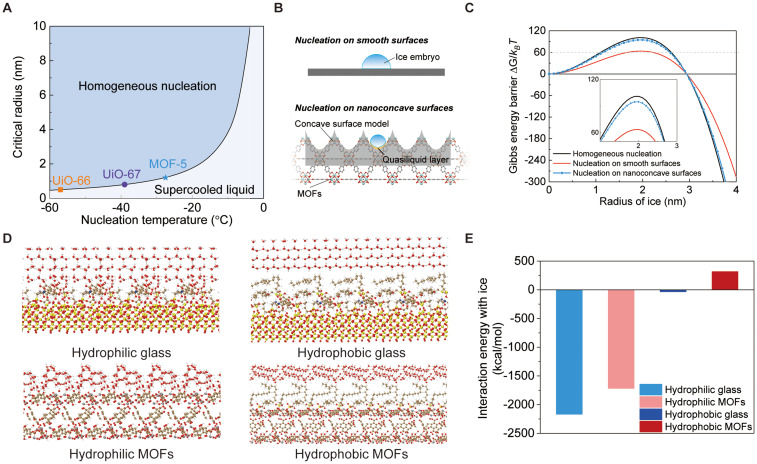
Classical nucleation theory (CNT) calculation and density functional theory (DFT) calculation results between ice and different surfaces. (A) Critical nucleation radius for homogeneous ice nucleation as a function of nucleation temperature. (B) Schematics of different heterogeneous nucleation models. (C) Calculated Gibbs free energy barriers for ice nucleation on different surfaces at −20 °C. (D) DFT-optimised models of ice interacting with four chemically distinct surfaces. Each model consists of a three-layer structure: the top layer represents a slab of hexagonal ice (ice I_h_), the middle layer contains molecular chains with different functional groups (*e.g.*, ATPES, organic linker, OTS, *etc*.), and the bottom layer represents the underlying substrate, either silica or a UiO-66–OH surface. (E) Calculated ice–surface interaction energies from DFT simulations, comparing MOF-based and glass-based surfaces with both hydrophilic and hydrophobic terminations.

Fig. 8A shows the critical radius for homogeneous ice nucleation as a function of nucleation temperature, overlaid with the pore sizes of different MOFs. According to classical nucleation theory (CNT), nucleation is only thermodynamically favourable when the critical radius is smaller than the pore size. However, this theoretical prediction clearly deviates from our experimental observations, as it does not account for the role of heterogeneous interfaces and the curvature effect. While the critical nucleus radius is the same for both homogeneous and heterogeneous nucleation, the associated Gibbs free energy barrier differs substantially, directly influencing nucleation probability and kinetics.

To address the above limitation, Fig. 8C presents the Gibbs free energy barriers for heterogeneous nucleation, as shown in the schematics in Fig. 8B. AFM-scale topography of MOF shows plenty of rough, convex structures (Fig. S4); however, we explicitly separate length scales: mesoscale particle features (radius >100 nm, see Fig. S5) are effectively flat up to a critical ice nucleus radius *r*_c_ ≈ 2.3–4.4 nm, whereas the intrinsic MOF porosity (radius ∼0.3 nm for UiO-66 window) imposes nanoconfinement. Thus, pores dominate the nucleation energetics while mesoscale convexity is negligible at the nucleation length scale. For UiO-66 (cubic crystal, *a* ≈ 2.07 nm), the nucleus spans ∼2 unit cells in diameter, reinforcing that the relevant curvature seen by the nucleus is set by sub-nanometre pores rather than >100 nm particle morphology. Therefore, we applied the heterogeneous nucleation equation, which accounts for the concave nanopores and the quasi-liquid layer effect at the ice–solid interface^57^ (see the SI for detailed calculations). Under conditions of −20 °C, on smooth surfaces (with an ice-water contact angle of 100°), the nucleation barrier is suppressed and found to be below 60*k*_B_*T*, suggesting that thermal fluctuations may overcome the barrier within experimental timescales. The Gibbs free energy barriers for nanoconcave surfaces (with the same contact angle and a solid fraction of 0.4 as UiO-type MOF^42^) are comparable to those for homogeneous nucleation, directly reflecting the nucleation-inhibiting effect of the nanoconfinement. Although this simple model does not capture molecular-level interactions, it offers an intuitive comparison framework that highlights the inhibitory role of MOF porosity on ice nucleation.

To gain further insight into interfacial interactions at the molecular level, we performed density functional theory (DFT) simulations (Fig. 8D) of ice in contact with four chemically distinct surfaces: a hydrophilic MOF (UiO-66–OH), its hydrophobically modified counterpart (UiO-66–OTS), and two glass-based analogues modelled as hydroxylated silica surfaces functionalised with APTES and linker molecules (hydrophilic) and further modified with OTS (hydrophobic). The calculated ice–surface interaction energies (Fig. 8E) are consistent with the experimental trends: regardless of surface chemistry (hydrophilic or hydrophobic), MOF surfaces consistently exhibit weaker interactions with ice than their glass counterparts. This directly supports our experimental findings that, even when wettability is similar, MOF-coated substrates show lower ice adhesion strengths (Fig. 7C) and reduced ice nucleation temperatures (Fig. 7B). The weaker interactions arise from the nanoscale pores on the MOF surface, which reduce the actual solid–ice contact area through the void effect,^42,78,81^ thereby increasing the energetic barrier to heterogeneous nucleation and facilitating easier ice detachment. Thus, the DFT results provide a molecular-level explanation for the enhanced icephobic performance observed experimentally.

### 3.6. Mechanical robustness of the MOF based coatings

In addition to demonstrating ice nucleation inhibition and reduced ice adhesion strength, the practical applicability of MOF-based icephobic coatings depends critically on their mechanical robustness and stability under low temperatures. UiO-type MOFs and MOF-5 were selected due to exceptional chemical and mechanical stability.^82^ Two durability tests were performed to evaluate substrate adhesion and the inherent mechanical stability of the surface-grown MOF films, including a standard tape-peel test (section 2.6) to assess the mechanical durability of the coating and cyclic icing/de-icing measurements to examine the retention of icephobic performance under freezing.

**Fig. 9:**
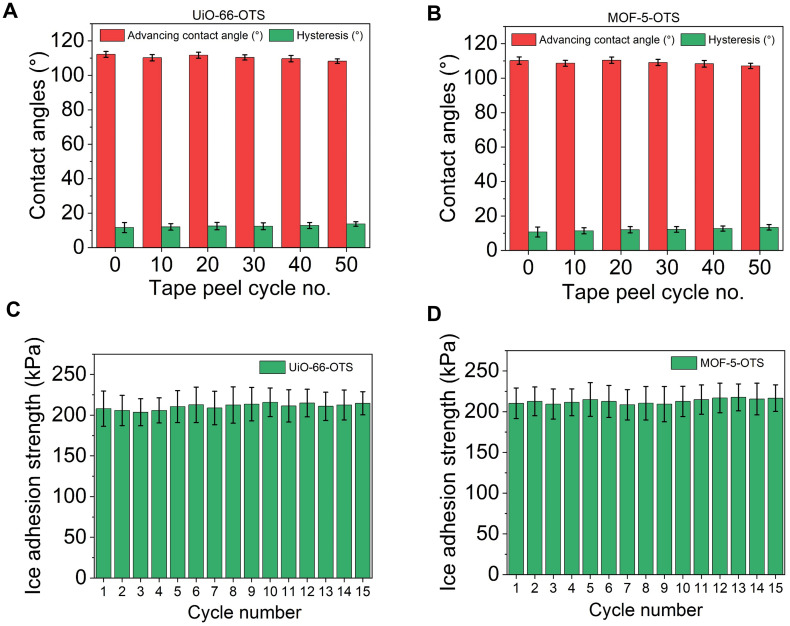
Robustness of surface-grown MOF coatings. Advancing water contact angle and contact angle hysteresis measured after successive tape-peel cycles for (A) UiO-66–OTS and (B) MOF-5–OTS. Ice adhesion strength recorded over 15 repeated icing/de-icing cycles for (C) UiO-66–OTS and (D) MOF-5–OTS.

During the tape-peel test, the advancing contact angle and contact angle hysteresis were measured after successive peeling cycles. Tape-peel tests were assessed on two OTS-functionalised MOF coatings, including UiO-66–OTS and MOF-5–OTS, as representatives of zirconium-based and zinc-based frameworks, respectively. Fig. 9A and B show that both UiO-66–OTS and MOF-5–OTS maintained a consistently high advancing contact angle and low hysteresis even after 50 tape-peel cycles, indicating good mechanical integrity and retention of surface functionality. In addition, the ice adhesion strength on UiO-66–OTS and MOF-5–OTS remained nearly unchanged over 15 consecutive freezing and detachment cycles, as shown in Fig. 9C and D. The preservation of low ice adhesion strength throughout the cycling tests confirms the durability of the MOF coatings. Taken together, these results corroborate the mechanical robustness of the nanohierarchical MOF coatings, suggesting they are promising candidates for durable icephobic applications.

## 4. Conclusions

In this study, we demonstrated that all selected surface-grown metal–organic framework (MOF) coatings effectively delayed ice nucleation and reduced ice adhesion strength. We found a geometric saturation regime: while sub-nanometre pores are crucial for disrupting ice nucleation, reducing the pore size beyond the critical ice nucleus radius provides no further enhancement. Once nanoconfinement is achieved, surface chemistry becomes the dominant tuning parameter. Consistent with this principle, hydrophobic functionalisation using OTS provided the most effective enhancement, yielding the lowest ice nucleation temperatures and the greatest reduction in ice adhesion strength. Supported by classical nucleation theory and molecular-level DFT simulations, our findings clarify the role of nanoporosity in icephobicity by demonstrating how the sub-nanometre pores decrease the actual solid-ice interfacial contact area, thereby increasing the energy barrier to nucleation and facilitating easier ice detachment. These insights represent a significant advancement in understanding how structure–property relationships in nanoporous coatings influence anti-icing performance. Validated by robust durability under cyclic tape peeling and icing/de-icing tests, these findings provide critical insights for designing robust, anti-icing surfaces.

## Author contributions

Simrandeep Bahal: methodology, investigation, visualization, validation, data curation, formal analysis, writing – original draft and review & editing, and conceptualization. Jianhui Zhang: writing – review & editing, software, methodology, visualization, data curation, formal analysis, and conceptualization. Vikramjeet Singh: writing – review & editing, methodology, supervision, and conceptualization. Prasenjit Kabi: writing – review & editing, methodology, and supervision. Abbas Heydari: writing – review & editing, methodology, and supervision. Manish K. Tiwari: writing – review & editing, supervision, project administration, funding acquisition, and conceptualization.

## Conflicts of interest

The authors declare no competing financial interest.

## Supplementary Material

NR-018-D5NR04825G-s001

## Data Availability

All the data supporting the findings and mentioned in this study are available in the main text and supplementary information (SI). Raw datasets are available from the corresponding author upon request for academic and non-commercial purposes. Supplementary information: material characterisation, classical nucleation theory for ice nucleation, ice nucleation and ice adhesion measurements, and DFT simulation, along with a representative force–time curve (Fig. S1), surface roughness (Fig. S2), AFM figures (Fig. S3–S7), SEM-EDS elemental mapping (Fig. S8 and S9), contact angle (Fig. S10), and FTIR spectra (Fig. S11). See DOI: https://doi.org/10.1039/d5nr04825g.
