# Supplementary material for: Tunable icephobicity of surface-grown metal–organic frameworks with nanohierarchical texture
Source: Nanoscale. 2026 Feb 23;18(14):7485–500. doi: 10.1039/d5nr04825g (PMC12974753; doi:10.1039/d5nr04825g)
Supplement: NR-018-D5NR04825G-s001 [file NR-018-D5NR04825G-s001.pdf]

This journal is © The Royal Society of Chemistry 2026

Supporting Information for

Tunable Icephobicity of Surface-Grown Metal-Organic Frameworks with Nanohierarchical Texture

Simrandeep Bahal[1,2†], Jianhui Zhang[1,2†], Vikramjeet Singh[3], Prasenjit Kabi[1,2], Abbas Heydari[1,2] and Manish K. Tiwari[1,2,3*]

[1]Nanoengineered Systems Laboratory, UCL Mechanical Engineering, University College London, London, WC1E 7JE, UK.

[2]UCL Hawkes Institute, University College London, London, W1W 7TS, UK

[3]Manufacturing Futures Lab, UCL Mechanical Engineering, University College London, London, E20 2AE, UK

***Corresponding author. Email: m.tiwari@ucl.ac.uk**

This file includes:

1. Supplementary Notes
2. Supplementary figures S1 to S11
3. Supplementary References

Supplementary Note 1. classical nucleation theory for ice nucleation

According to classical nucleation theory (CNT), for homogeneous ice nucleation, when a spherical nucleus of radius r forms in a supercooled liquid, the total Gibbs free energy change $\Delta G_{hom}(r)$ is expressed as:

$$\Delta G_{hom}(r) = 4\pi r^2 \gamma + \frac{4}{3}\pi r^3 \Delta G_{f,v}$$

(1)

where γ is the ice-water interfacial energy, $\Delta G_{f,v}$ is volume free energy difference per unit volume. To find the critical radius r_c, we take the derivative of $\Delta G_{hom}(r)$ and set it to zero. The Gibbs free energy of formation of a critical ice cluster can be obtained as[1-3] :

$$\Delta G^* = \frac{16\pi\gamma^3}{3(\Delta G_{f,v})^2} \qquad (2)$$

Thus, the critical radius of ice embryo r_c is

$$r_c = \frac{2\gamma}{\Delta G_{f,v}} \qquad (3)$$

The volume free energy difference is given by:

$$\Delta G_{f,v} = \frac{\Delta H_f (T_m - T)}{T_m v_m} \qquad (4)$$

where ΔH_f is the enthalpy of fusion of ice, T_m is melting point of ice, i.e. 273.15 K, T is the actual system temperature, v_m is molar volume of ice. The critical radius of ice embryo was calculated as $r_c = 4.4\ nm$ and $r_c = 2.3\ nm$ at -10 °C and -20 °C respectively.

For heterogeneous nucleation, which occurs at interfaces such as solid surfaces or impurities, the critical radius is formally the same as that in homogeneous nucleation. However, the nucleation energy barrier is reduced in heterogeneous nucleation due to the presence of a substrate. This reduction can be described by a geometric correction factor $f(\theta)$ and solid fraction ϕ_s, which depends on the contact angle θ between the ice and water:

$$\Delta G_{het}(r) = \Delta G_{hom}(r) \cdot \left[f(\theta) + (1 - \phi_s)(1 - f(\theta)) \right] \qquad (5)$$

with:

$$f(\theta) = \frac{1}{4}(2 + \cos\theta)(1 - \cos\theta)^2 \qquad (6)$$

This is a hybrid model that incorporates both heterogeneous (flat solids) and homogeneous nucleation (holes) contributions by considering the rough surface as a composite of solid

regions that promote heterogeneous nucleation and void regions where homogeneous nucleation dominates.

We further account for porosity-dominated heterogeneous nucleation model with the nanoconcave features, originating from MOF porous surfaces. The intrinsic pores of the MOF dominate the confinement effect, as their dimensions are close to the critical ice nucleus size. At such small length scales, the quasi-liquid layer at the ice–solid interface couples with this nanoporosity to give rise to an effective nanoscale confinement effect that elevates the nucleation barrier. The nucleation energy barrier can be expressed as[4]:

$$\Delta G_{het}(r) = \Delta G_{hom}(r) \cdot \left[f(m,R) + (1 - \phi_s)(1 - f(m,R)) \right] \quad (7)$$

with:

$$f(m,R) = \frac{1}{2}\left\{1 - \left(\frac{1 + mx}{g}\right)^3 - x^3\left[2 - 3\left(\frac{x + m}{g}\right) + \left(\frac{x + m}{g}\right)^3\right] + 3mx^2\left(\frac{x + m}{g} - 1\right)\right\} \quad (8)$$

$$g = (1 + 2mx + x^2)^{0.5} \quad (9)$$

$$x = R/r_c \quad (10)$$

$$m = (\cos\theta + 1)e^{-\frac{d}{\xi}} - 1 \quad (11)$$

where R, d, and ξ are the roughness curvature radius, the thickness of the quasi-liquid layer, and the decay length of the interaction with the bulk. r_c stays the same for both homogeneous and heterogeneous ice nucleation, which is calculated as 2.3 nm at -20 °C, as mentioned above. d is estimated as 0.1 nm through the linearized Gibbs-Thomson relation as reported in the literature[4].

The formation of a critical nucleus is treated as a thermally activated barrier-crossing process. The probability of a nucleation event (P) occurring is governed by the competition between thermal fluctuations and the free energy barrier associated with forming a stable nucleus. The likelihood of nucleation is expressed by an Arrhenius-type equation:

$$P \propto exp(-\frac{\Delta G(r)}{k_B T}) \quad (12)$$

where k_B is the Boltzmann constant. When $\Delta G > 60\ k_B T$, nucleation is exceedingly rare.

Supplementary Note 2. Ice nucleation and ice adhesion measurements

The ice nucleation temperature and ice adhesion strength were measured using a custom designed benchtop setup. The setup consisted of a Peltier element (Laird, 387004726), mounted on top of a tubed plate (Laird, ATS-TCP-1001), and a four-pass copper tube heat exchanger. The Peltier element was powered with the help of a power supply (PS-24-25, Tetech INC) and a microcontroller (TC-720, Tetech INC). As the Peltier element was powered, heat dissipated

at the hot side of Peltier was removed continuously by pumping a mixture of cold ethylene glycol solution and water through the heat exchanger with the help of a chilling unit (Julabo FP52-SL). A cooling rate between 1 and 5 K/min on the Peltier element was controlled using a microcontroller and a chilling unit. During the experiments, the substrate was placed in thermal contact with the Peltier. A Type K thermocouple (RS Pro) was mounted on the Peltier element to monitor and log its temperature during the experiments. This thermocouple was connected to the computer *via* a thermocouple DAQ (NI-DAQ-9174). A LabVIEW program was written to measure and log the temperature in an excel file. The entire Peltier and heat exchanger assembly was enclosed inside a 3D-printed chamber. An acrylic sheet, fixed in the top lid of this chamber allowed for visual access of the droplets during the experiments. The relative humidity inside the chamber was monitored using a humidity sensor (HC2A-S3 Rotronic) and a webcam (Logitech-C920 HD) was used to record the images of droplets during the experiments.

Nucleation temperature measurements: During the nucleation temperature experiments, the substrate was placed on the Peltier element and around 30 droplets each of 2 microlitre volume were pipetted onto the substrate at room temperature. A very gentle flow of dry nitrogen was passed through the box to prevent frost formation during the experiment. The temperature of the substrate was then reduced from room temperature at a cooling rate of 1°C/min till all the droplets were frozen. A movie of this cooling process was recorded with the help of webcam to detect the individual freezing of the droplets. The images were recorded at a frequency which depended upon the experimental cooling rate. Hence, the temperature of the substrate at each frame was known. Three trials of nucleation temperature measurement with 30 droplets were performed for each substrate. Frozen droplets could be easily distinguished from the liquid droplets as they appeared white in the images due to increased light scattering. The information obtained from the images was used to determine the fraction of frozen droplets f_{ice} as a function of temperature. The fraction of frozen droplets was computed by the equation $f_{ice} = n_{ice}(T)/n_{tot}$ where $n_{ice}(T)$ represented the number of water droplets that had frozen at a specific temperature T and n_{tot} was the total number of water droplets pipetted onto the substrate.

Ice adhesion experiments: After placing the substrate on the Peltier element, a cylindrical cuvette block of 8 mm inner diameter and 11 mm outer diameter was placed on top of the substrate. This cuvette block was filled with water, and the temperature of the substrate was brought down to -20 °C and was maintained at this temperature for an hour till water in the cuvette had frozen completely to form ice. A force was applied parallel to the substrate on the outer surface of the cuvette with the help of a force gage (Mark-10 Model M4-20) which was connected to a metallic rod that pushed the cuvette block, and the applied force was recorded by the gage. This force gage was moved towards the ice filled cuvette block at a constant velocity of 0.02 mm/sec with the help of a custom-built stepper motor controller. The peak force (F) was recorded from the force vs time curve, and the ice adhesion strength (τ) was calculated using the equation $\tau = F/A$ where A is the base area of the cuvette. The average ice adhesion strength was obtained from at least three independent measurements on different samples.

Supplementary Note 3. DFT simulation on ice/surfaces interactions

Density functional theory (DFT) calculations were performed using the CP2K software package to investigate the interfacial interactions between ice and four different surfaces: linker-coated glass, UiO-66-OH, OTS-coated glass, and UiO-66-OTS. All simulations were carried out using the Quickstep module in CP2K. All DFT calculations employed the DZVP-MOLOPT-SR-GTH Gaussian basis set in combination with GTH-PBE pseudopotentials, along with Grimme's D3 dispersion correction to account for van der Waals interactions.

Surface models were constructed based on either crystalline UiO-66 slabs or amorphous silica surfaces functionalised with the corresponding linker or silane groups. A hexagonal ice Ih bilayer was placed on each surface model, and the interface was fully relaxed to determine the equilibrium adsorption geometry. Periodic boundary conditions were applied in all directions with a vacuum gap of at least 60 Å in the z-direction to avoid spurious interactions between periodic images.

The interaction energy between ice and each surface was computed as[5]:

$$E_{int} = E_{tot} - E_{ice} - E_{surface} + E_{tot}^{BSSE}$$

(13)

To account for basis set superposition error (BSSE), the counterpoise correction method was applied following the Boys–Bernardi scheme. Specifically, ghost atoms were introduced in the calculations of the isolated subsystems (ice and surface), ensuring that the basis functions of both interacting partners were included in each energy evaluation. These BSSE-corrected interaction energies provide more accurate insights into how surface chemistry and porosity influence ice–surface interactions relevant to icephobic behaviour.

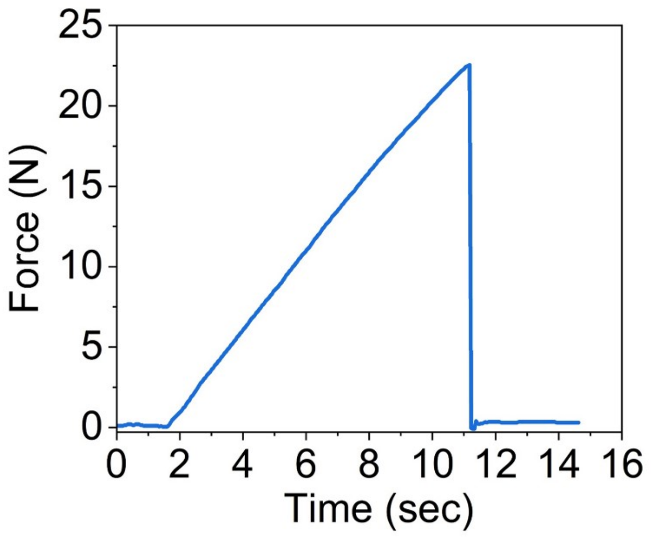

Figure S1. Representative force-time curve recorded during the ice adhesion measurement on a MOF-coated substrate. The peak force corresponds to the maximum shear force required to initiate ice detachment from the substrate. Ice adhesion strength (τ) was calculated as $\tau = F/A$, where F is the peak detachment force and A is the contact area between the ice column (8 mm inner diameter) and the substrate.

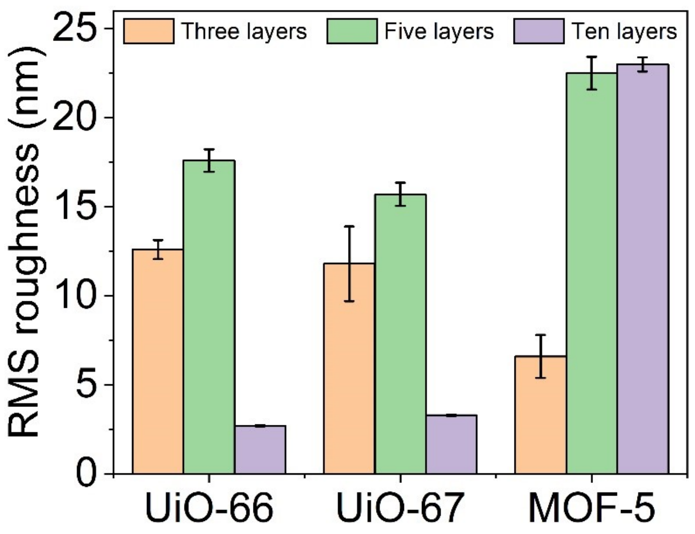

Figure S2: Root mean square (RMS) surface roughness values of UiO-66, UiO-67 and MOF-5 films as measured by AFM for three, five and ten-layer configurations. The plot illustrates the increase in roughness from three to five layers for all the three MOFs, followed by a significant reduction in roughness for UiO-66 and UiO-67 at ten layers and a sustained high roughness for MOF-5 due to a continued crystallite growth. The rms roughness of bare glass substrate was around 0.54 nm.

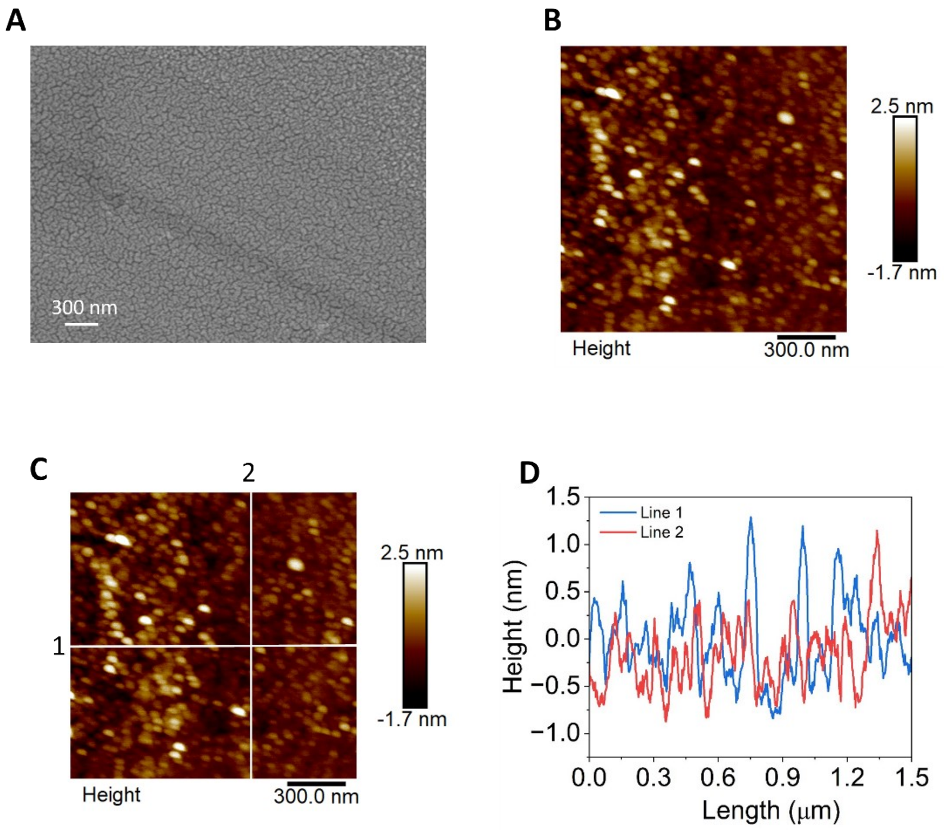

Figure S3: Surface morphology of the bare (uncoated) glass assessed using SEM imaging and AFM scans. This bare glass substrate was used as a reference to compare with various MOF substrates. (A) SEM image showing the surface morphology of the bare glass. (B) Corresponding AFM topography image revealing surface features. (C) AFM image indicating two perpendicular white lines (marked as lines 1 and 2) along which height profiles were measured. (D) Cross-sectional height profiles along lines 1 and 2 from (C) illustrating the relatively smooth surface of the bare glass.

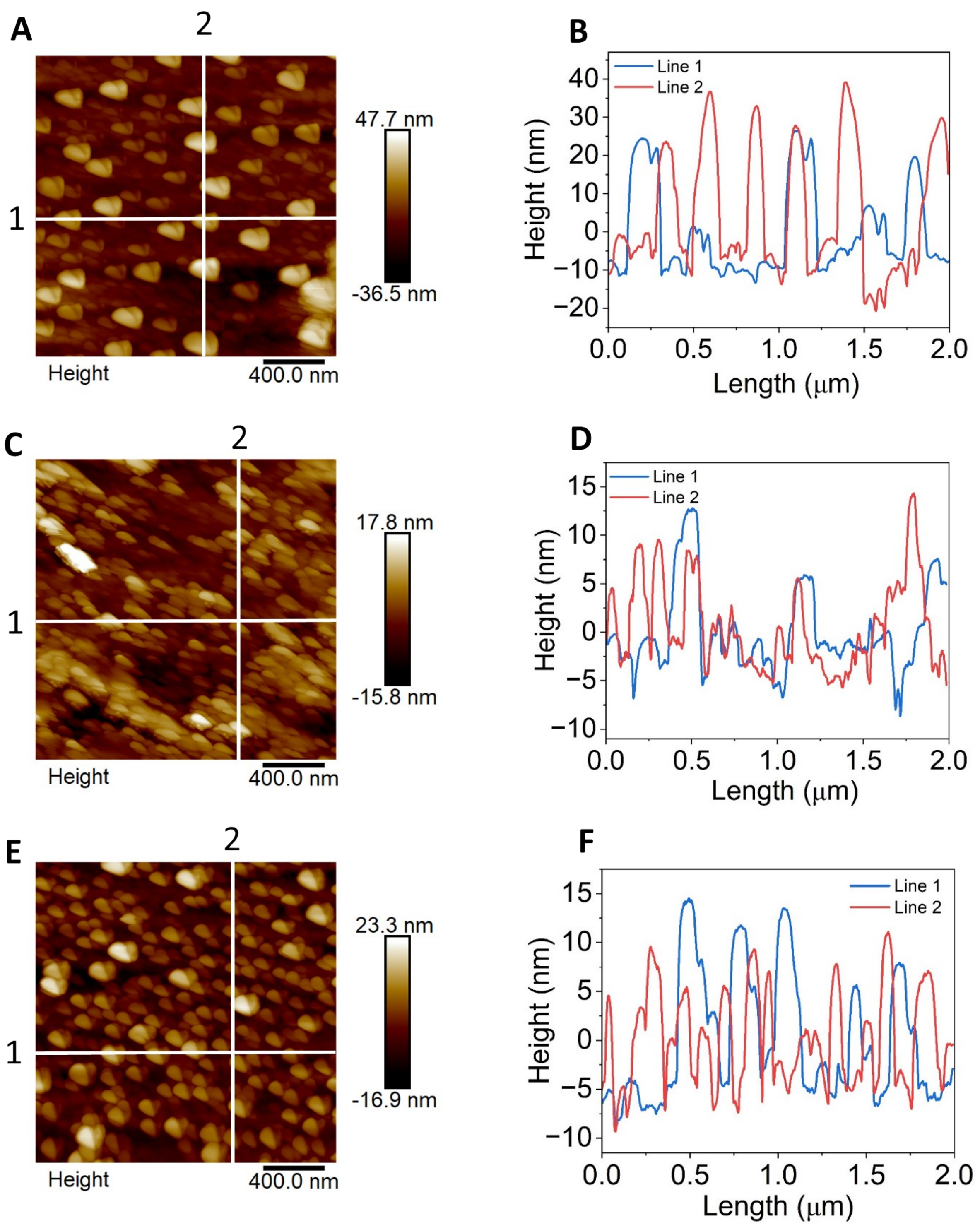

Figure S4: AFM topographical analysis for MOF-coated substrates when the number of layers were three. (A, B) UiO-66; (C, D) UiO-67; and (E, F) MOF-5. Left column (A, C, E): AFM topography images showing two perpendicular white lines (marked as lines 1 and 2), along which cross-sectional height profiles were recorded. Right column (B, D, F): Corresponding height profiles demonstrating the surface morphology and roughness at the three-layer configuration where discrete surface features were observed as indicative of initial stage of MOF growth.

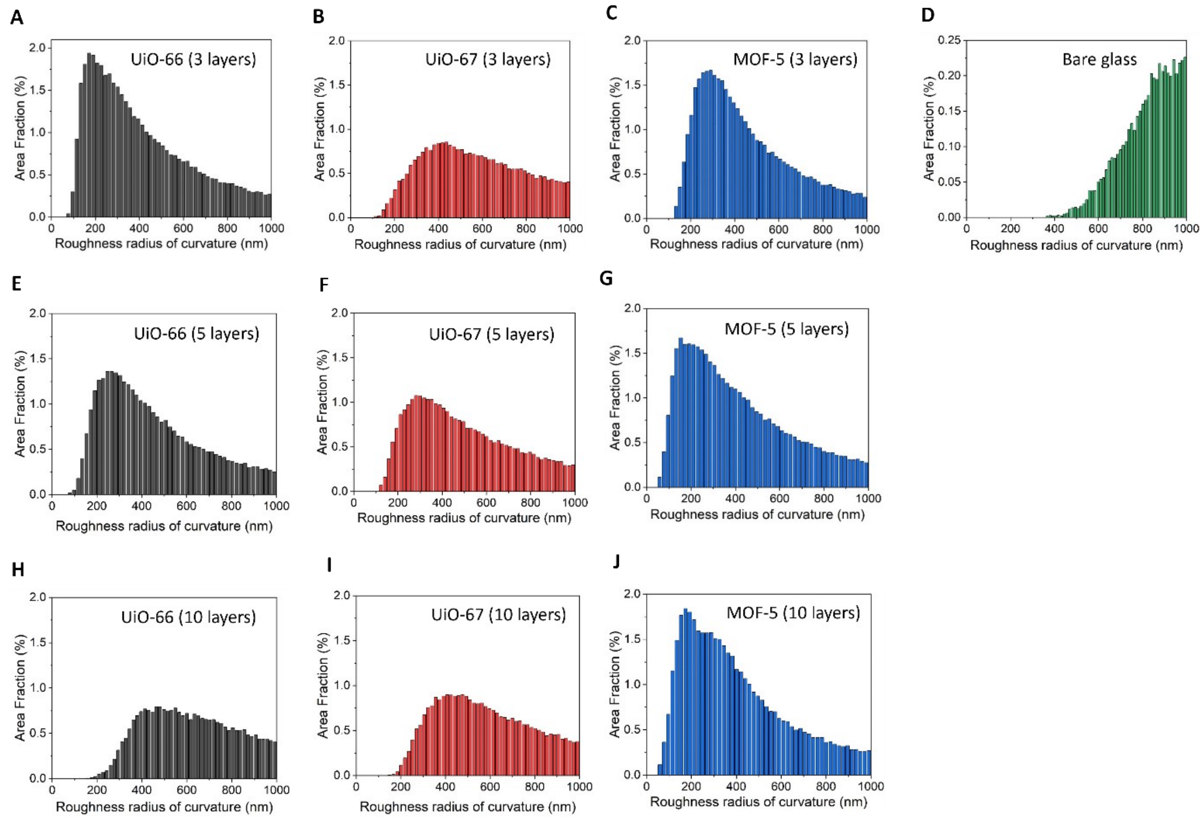

Figure S5: Area fraction occupied by nanopits plotted as a function of their roughness radius of curvature for the various substrates as extracted from their AFM topography scans. (A–C) correspond to UiO-66, UiO-67 and MOF-5 coated substrates respectively at the three-layer configuration whereas (D) correspond to bare glass for reference. (E–G) correspond to UiO-66, UiO-67 and MOF-5 coated substrates respectively at the five-layer configuration and (H–J) depict the corresponding distributions at ten layers. Compared to the smoother reference surface of bare glass (D), the MOF-coated substrates displayed significantly higher density of nanopits and comparatively smaller radii of curvature which highlighted their distinct nanohierarchical surface morphologies. Notably, the roughness radius of curvature on every MOF coated substrate exceeded the critical threshold of $10r_c$ where r_c is the critical ice embryo radius (2.3 – 4.4 nm for the temperature range examined), thus $10r_c$ falling within the range of approximately 23 – 44 nm).

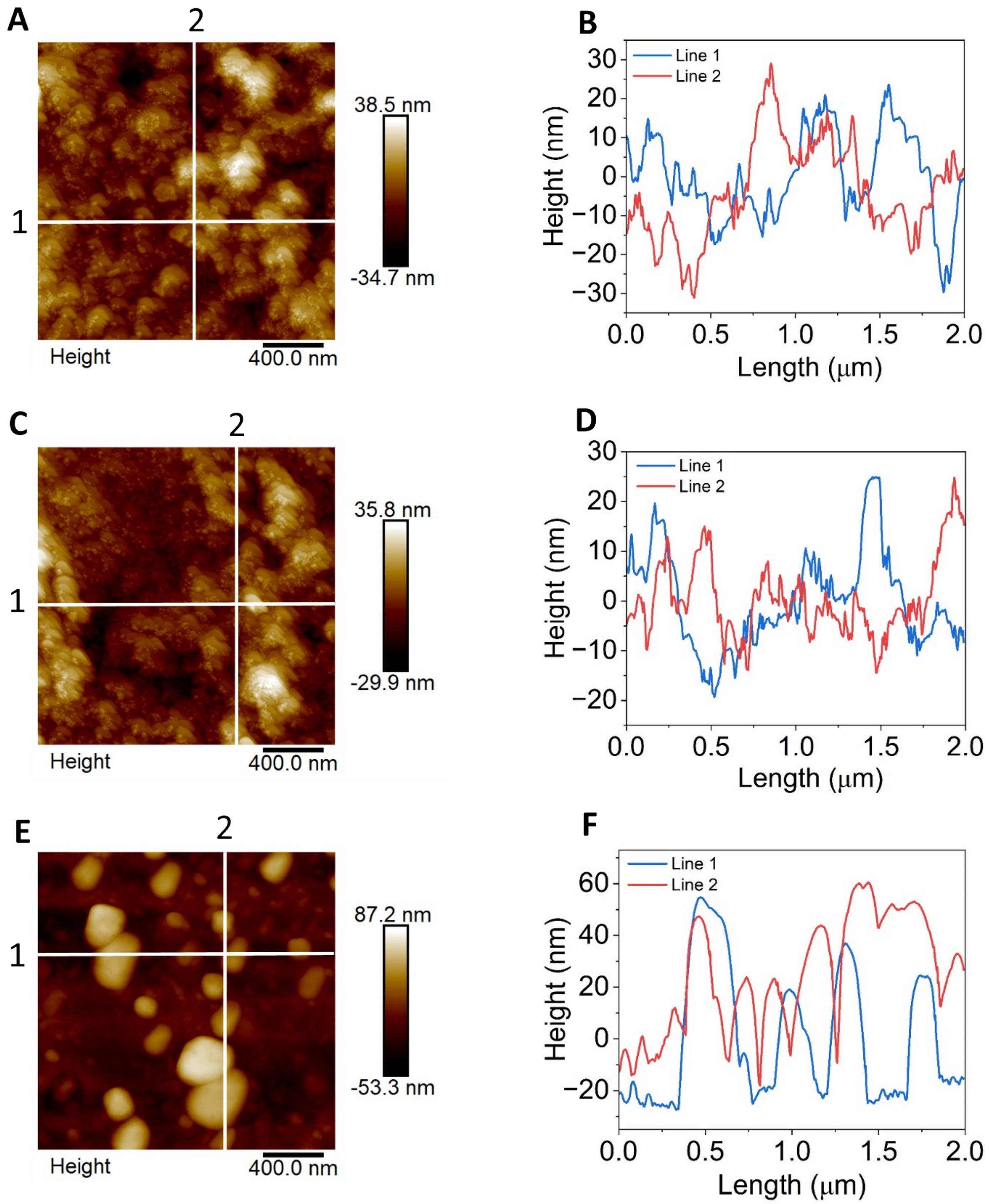

Figure S6: AFM topographical analysis for MOF-coated substrates when the number of layers were five. (A, B) UiO-66; (C, D) UiO-67; and (E, F) MOF-5. Left column (A, C, E): AFM topography images showing two perpendicular white lines (marked as lines 1 and 2), along which cross-sectional height profiles were recorded. Right column (B, D, F): Corresponding height profiles illustrating the increased surface roughness and interconnected particle morphology at the five-layer configuration.

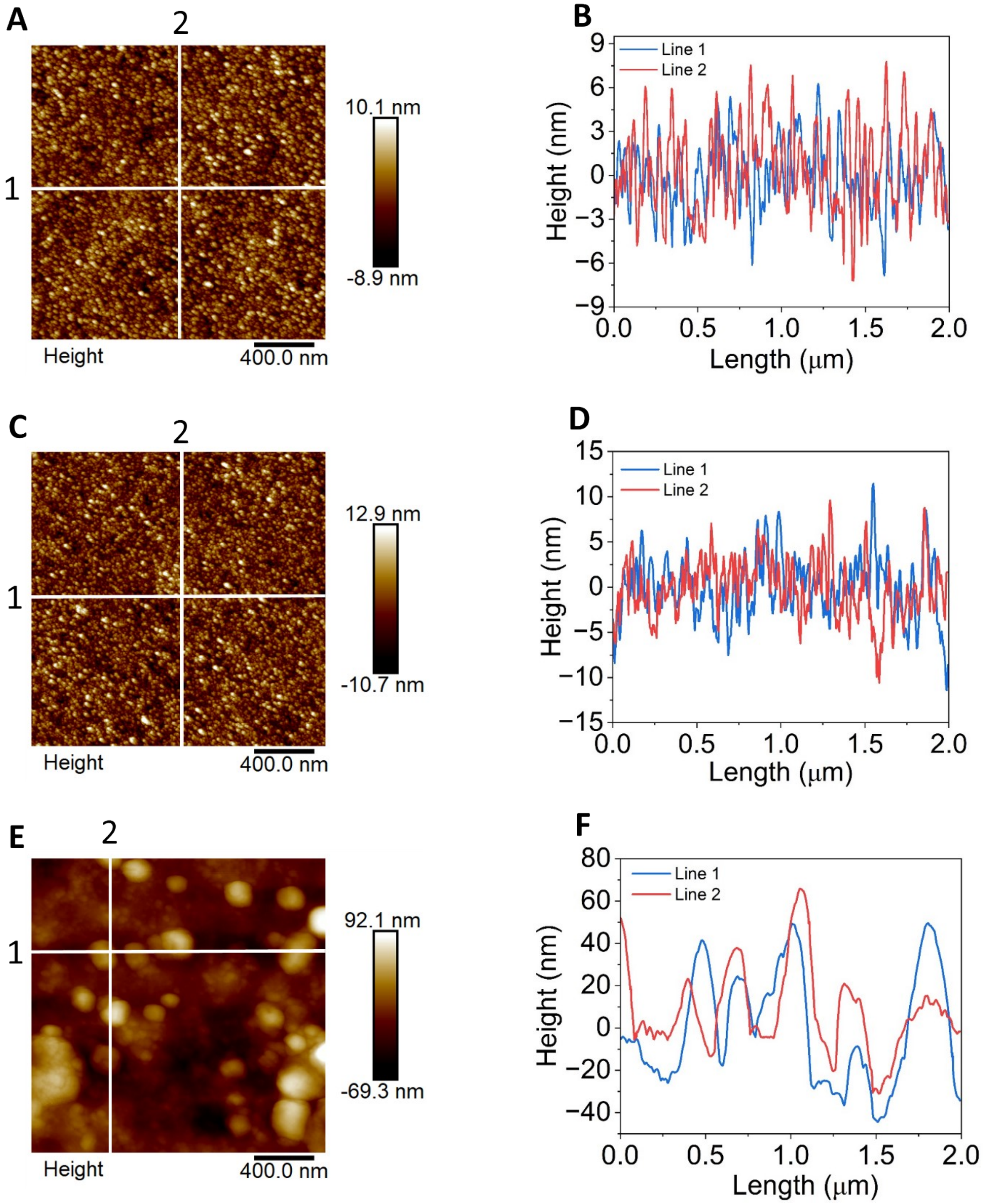

Figure S7: AFM topographical analysis for MOF-coated substrates when the number of layers were ten. (A, B) UiO-66; (C, D) UiO-67; and (E, F) MOF-5. Left column (A, C, E): AFM topography images showing two perpendicular white lines (marked as lines 1 and 2), along which cross-sectional height profiles were recorded. Right column (B, D, F): Corresponding height profiles showing the surface morphology at the ten-layer configuration highlighting smoother, continuous surfaces for UiO-66 and UiO-67 and prominent cubic crystallites for MOF-5.

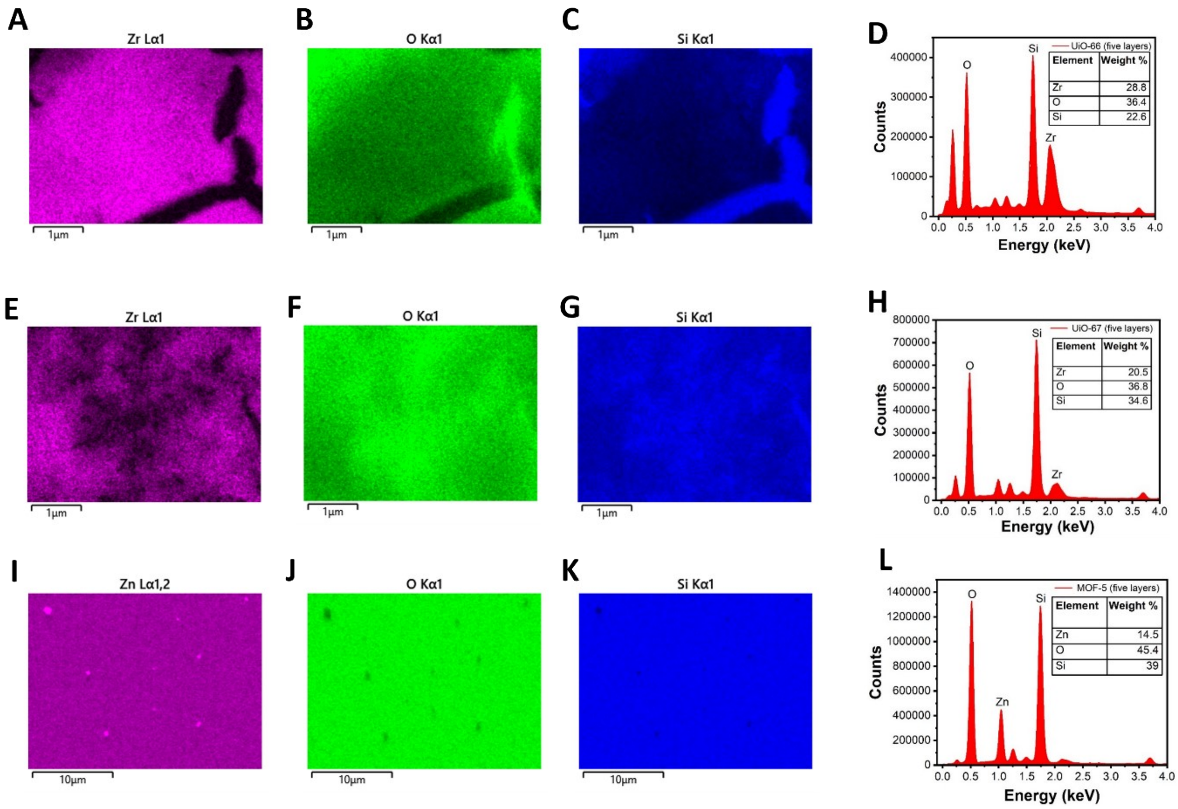

Figure S8: SEM-EDS elemental mapping of surface-grown MOF films (pristine UiO-66, UiO-67, and MOF-5) on glass substrates (five-layer configuration). The top row (A–D) corresponds to UiO-66, the middle row (E–H) corresponds to UiO-67, and the bottom row (I–L) corresponds to MOF-5. Columns show elemental distribution maps of Zirconium (Zr) for UiO-66 and UiO-67 or Zinc (Zn) for MOF-5, Oxygen (O), and Silicon (Si) followed by corresponding EDS spectra (counts vs. energy in keV). The uniform distribution of Zr and Zn confirmed the successful MOF synthesis across the substrate. The Si signal arose predominantly from the underlying glass substrate and was therefore expected in all samples.

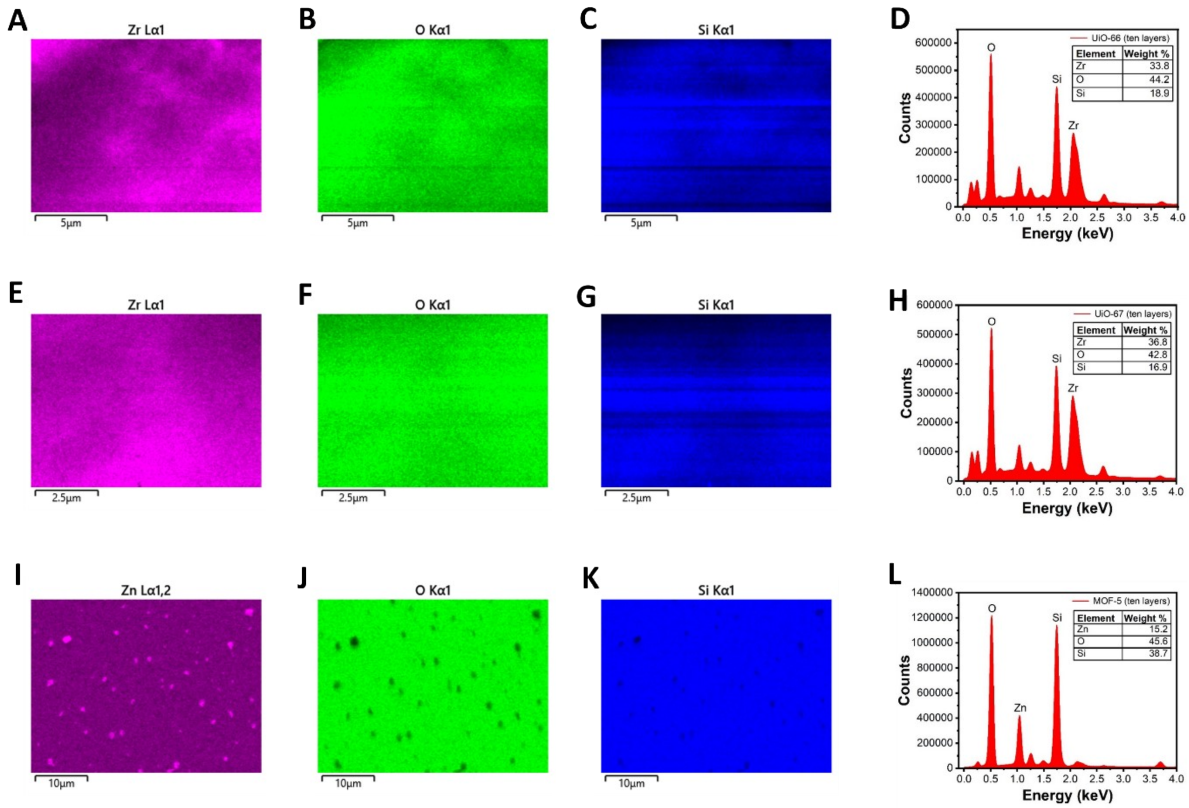

Figure S9: SEM-EDS elemental mapping of surface-grown MOF films (pristine UiO-66, UiO-67, and MOF-5) on glass substrates (ten-layer configuration). The top row (A–D) corresponds to UiO-66, the middle row (E–H) corresponds to UiO-67, and the bottom row (I–L) corresponds to MOF-5. Columns show elemental distribution maps of Zirconium (Zr) for UiO-66 and UiO-67 or Zinc (Zn) for MOF-5, Oxygen (O), and Silicon (Si) followed by corresponding EDS spectra (counts vs. energy in keV). The uniform distribution of Zr and Zn confirmed the successful MOF synthesis across the substrate. The Si signal arose predominantly from the underlying glass substrate and was therefore expected in all samples.

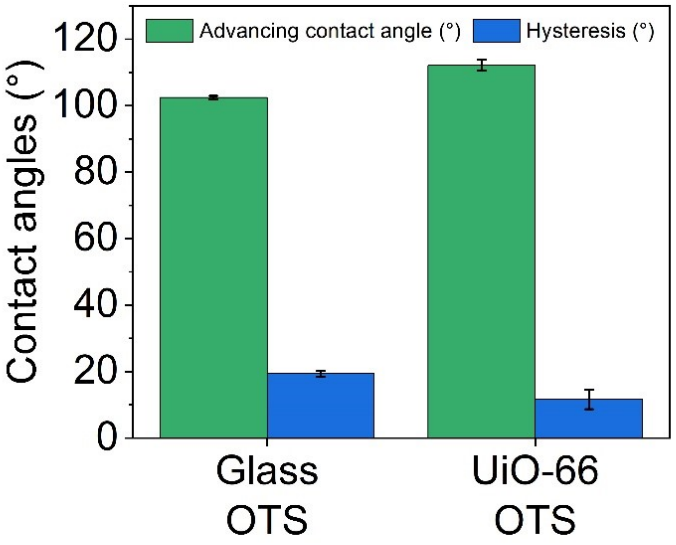

Figure S10. Advancing contact angle and contact angle hysteresis values measured on OTS-modified bare glass and UiO-66-OTS substrate.

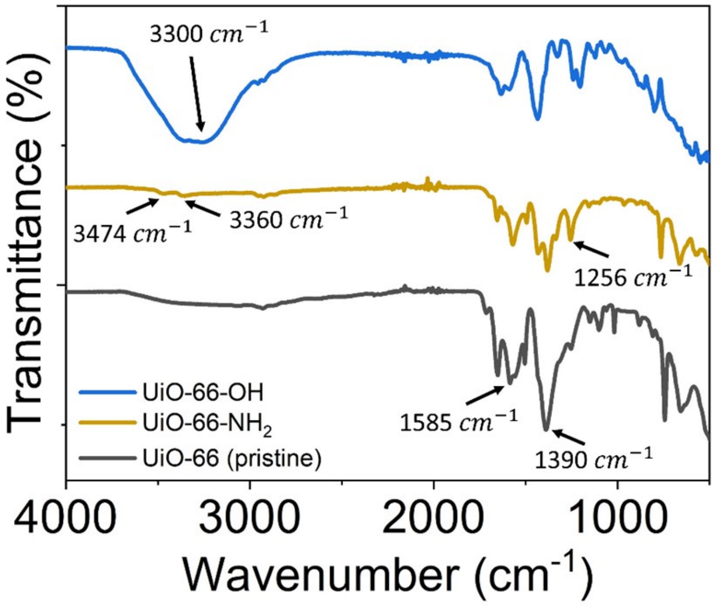

Figure S11: FTIR spectra of pristine UiO-66, UiO-66-OH, and UiO-66-NH_2. All spectra display the characteristic carboxylate bands of the UiO-66 framework [asymmetric ν(COO^-) ≈ 1585 cm^{-1}; symmetric ν(COO^-) ≈ 1390 cm^{-1}] and the Zr–O cluster vibrations around 700 cm^{-1}, confirming that the UiO-66 framework remained intact in all variants. UiO-66-OH shows a broad O–H stretching band centred at ~ 3200 – 3400 cm^{-1}, indicative of introduced hydroxyl groups. UiO-66-NH_2 exhibits N–H stretching bands at 3500 – 3300 cm^{-1} and a C–N stretching band of the amide bond at ~1256 cm^{-1}, confirming successful incorporation of the amine functionality.

References

(1) Pruppacher, H. R.; Klett, J. D.; Wang, P. K. Microphysics of clouds and precipitation. Taylor & Francis: 1998.

(2) Mullin, J. W. *Crystallization*; Elsevier, 2001.

(3) Ickes, L.; Welti, A.; Hoose, C.; Lohmann, U. Classical nucleation theory of homogeneous freezing of water: thermodynamic and kinetic parameters. *Physical Chemistry Chemical Physics* **2015**, *17* (8), 5514-5537.

(4) Eberle, P.; Tiwari, M. K.; Maitra, T.; Poulikakos, D. Rational nanostructuring of surfaces for extraordinary icephobicity. *Nanoscale* **2014**, *6* (9), 4874-4881.

(5) Vilela Oliveira, D.; Laun, J.; Peintinger, M. F.; Bredow, T. BSSE-correction scheme for consistent gaussian basis sets of double-and triple-zeta valence with polarization quality for solid-state calculations. *Journal of Computational Chemistry* **2019**, *40* (27), 2364-2376.
